# Supplementary material for: CORE GREML for estimating covariance between random effects in linear mixed models for complex trait analyses
Source: Nat Commun. 2020 Aug 21;11:4208. doi: 10.1038/s41467-020-18085-5 (PMC7442840; doi:10.1038/s41467-020-18085-5)
Supplement: Supplementary file 1 — Supplementary Information [file 41467_2020_18085_MOESM1_ESM.pdf]

Supplementary Information

CORE GREML for estimating covariance between random effects in linear mixed models for complex trait analyses

Zhou et al.

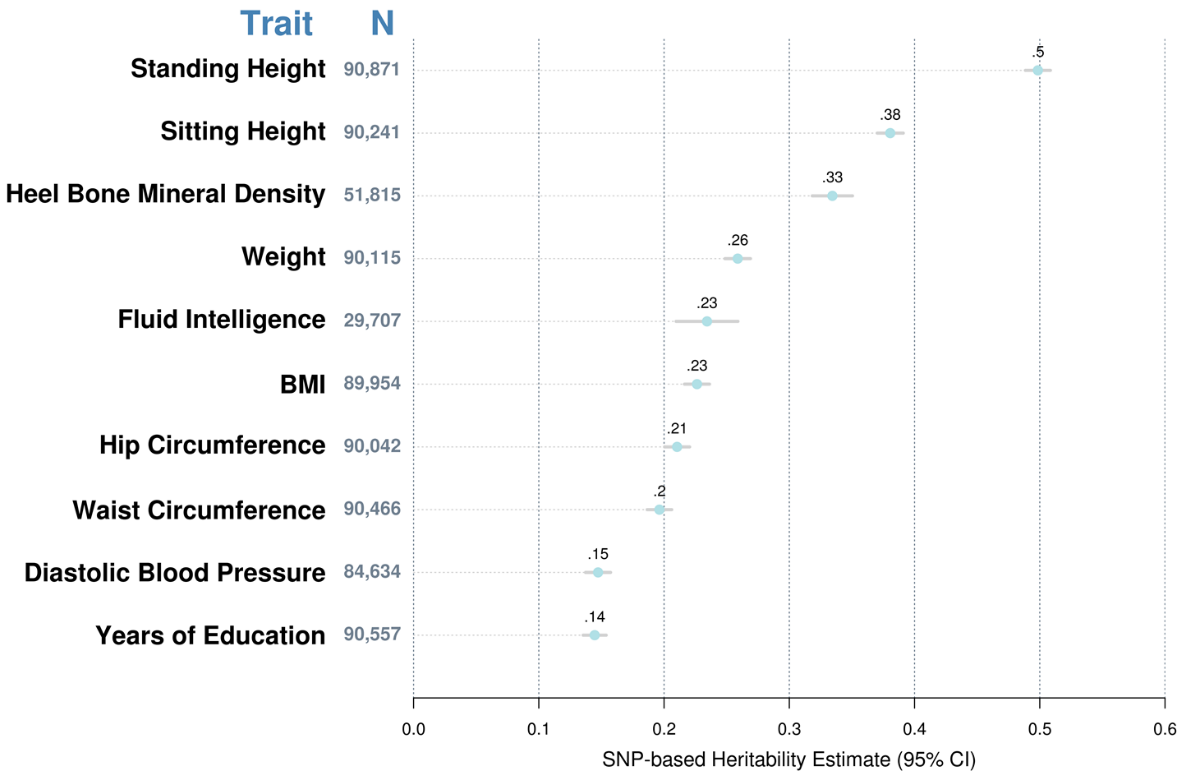

Supplementary Figure 1. SNP-based heritability estimates. Error bars are 95% confidence intervals (based on s.e.m). N = sample size. Source data are provided as a Source Data file.

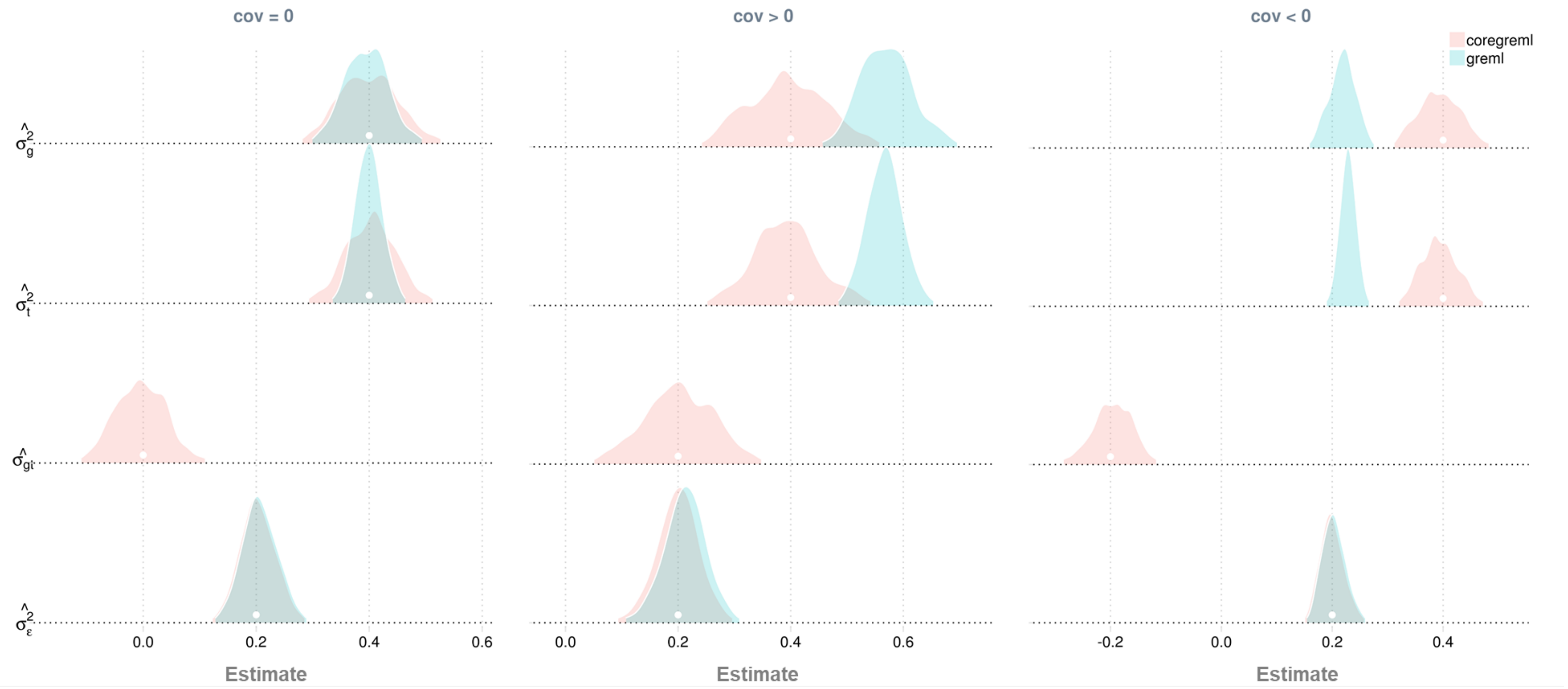

Supplementary Figure 2. Sampling distributions of model parameters for the genome-transcriptome partitioning model. Five-hundred replicates of phenotypic data (*n* = 10,000) were simulated under each of three parameter settings, where the covariance between the random effects of the genome and the imputed transcriptome was zero (cov=0), positive (cov>0) and negative (cov<0). For each replicate, model parameters were estimated by the traditional method, i.e., GREML, and the proposed method, i.e., CORE GREML. $\widehat{\sigma_g^2}$ = estimated phenotypic variance explained by the genome; $\widehat{\sigma_t^2}$ = estimated phenotypic variance explained by the imputed transcriptome; $\widehat{\sigma_{gt}}$ = estimated covariance between random effects of the genome and those of the imputed transcriptome; and $\widehat{\sigma_\varepsilon^2}$ = estimated residual variance. True values of model parameters are in dots. Source data are provided as a Source Data file.

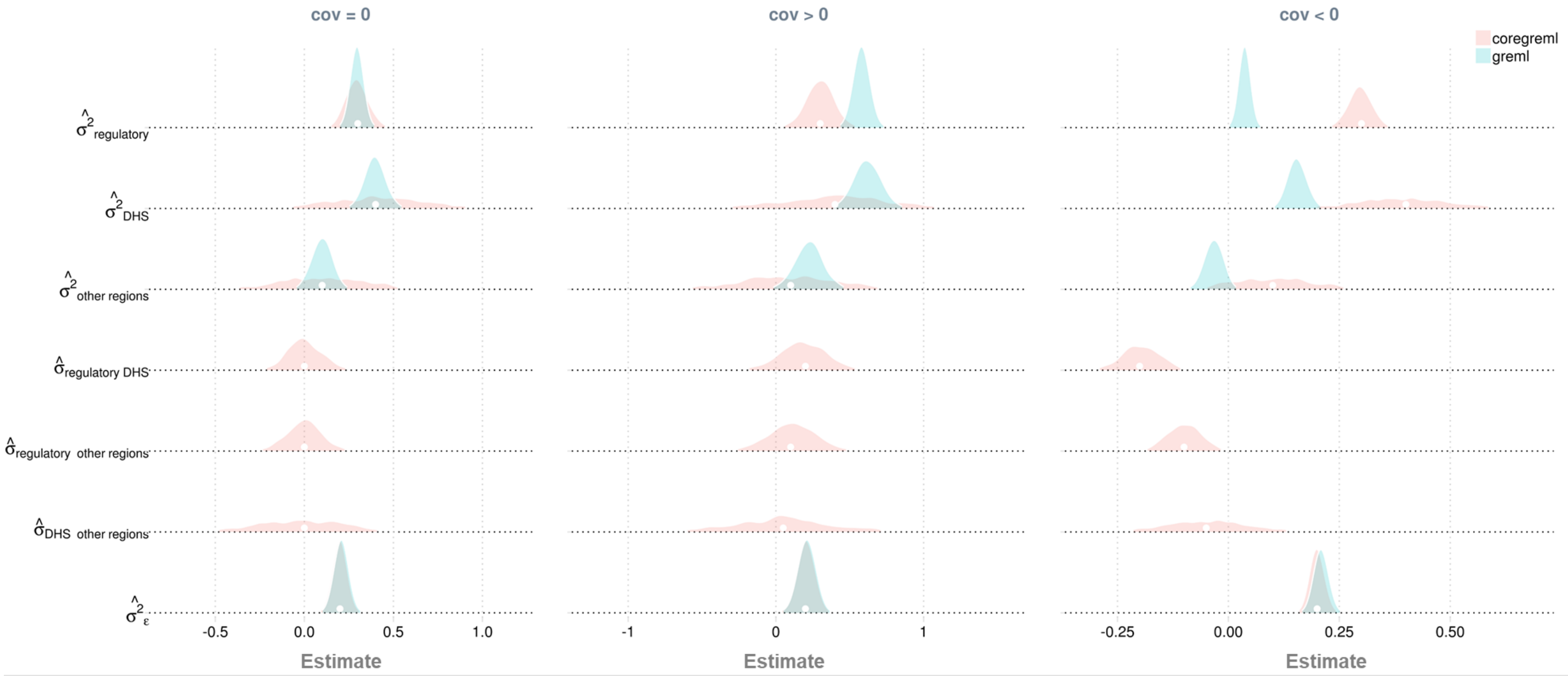

Supplementary Figure 3. Sampling distributions of model parameters for the genomic partitioning model. Five-hundred replicates of phenotypic data (n = 10,000) were simulated under each of three parameter settings, where covariances between the random effects of three functional regions of the genome were zero (cov=0), positive (cov>0) and negative (cov<0). The three functional regions of the genome are regulatory regions, DNase I hypersensitivity sites (DHS), and all other regions. For each replicate, model parameters were estimated using the traditional method, i.e., GREML, and the proposed method, i.e., CORE GREML. $\widehat{\sigma^2}_{\text{regulatory}}$, $\widehat{\sigma^2}_{\text{DHS}}$ and $\widehat{\sigma^2}_{\text{other regions}}$ denote estimated phenotypic variances explained by the three functional regions; $\widehat{\sigma_{\varepsilon}^2}$ denotes estimated residual variance; $\hat{\sigma}_{\text{regulary DHS}}$, $\hat{\sigma}_{\text{regulary other regions}}$ and $\hat{\sigma}_{\text{DHS other regions}}$ denote estimated covariances between random effects of these functional regions. True values of model parameters are in dots. Source data are provided as a Source Data file.

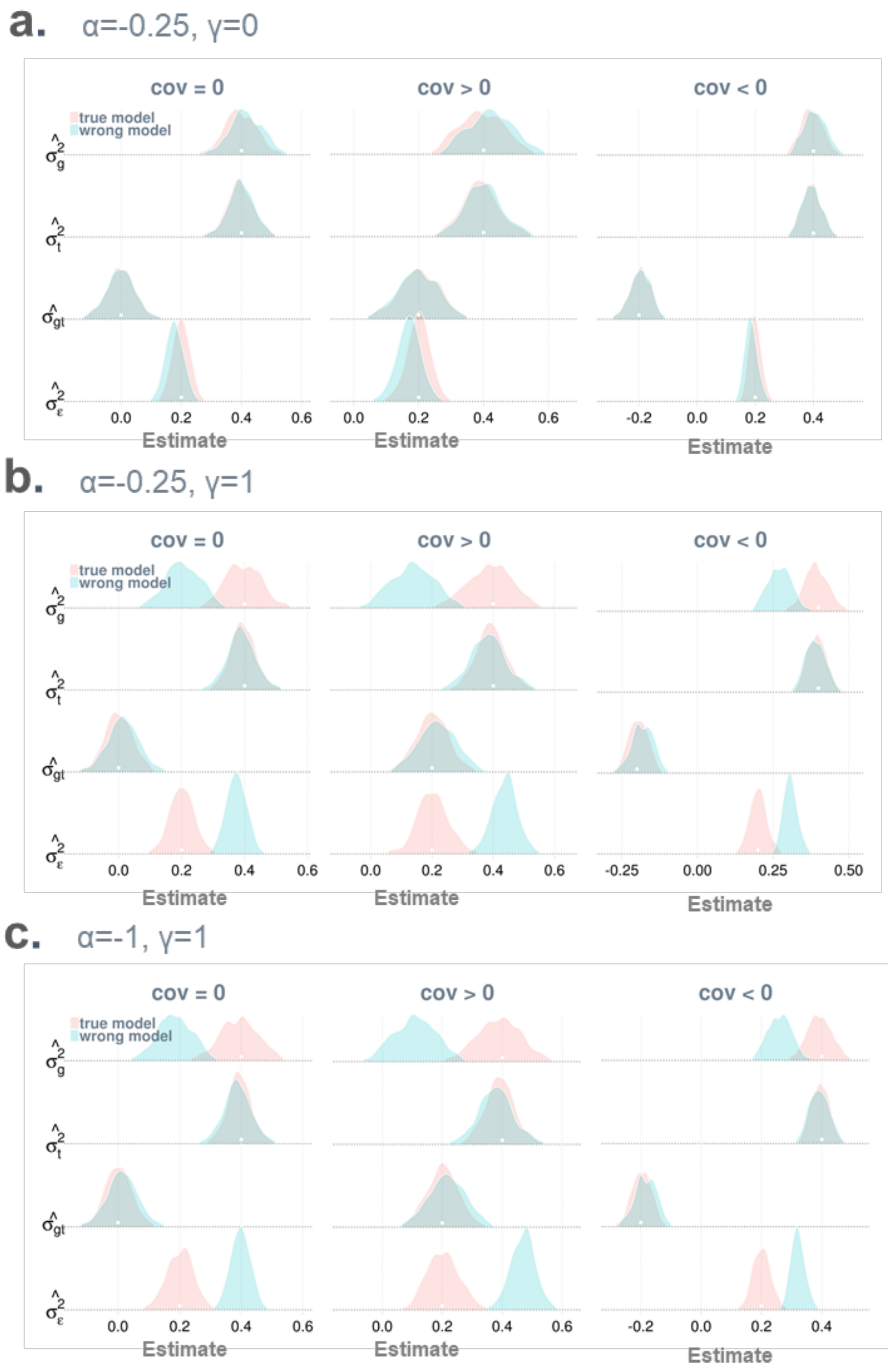

Supplementary Figure 4. Misspecification of genetic architecture in the estimation model affects variance estimates but not covariance estimates by CORE GREML. Shown in each panel are sampling distributions of model parameters by CORE GREML when the estimation model assumes the correct genetic architecture of the simulated trait (i.e., the true model is fitted) and when the estimation model mis-specifies the genetic architecture (i.e., a wrong model is fitted). $\widehat{\sigma_g^2}$ = estimated phenotypic variance explained by the genome; $\widehat{\sigma_t^2}$ = estimated phenotypic variance explained by the imputed transcriptome; $\widehat{\sigma_{gt}}$ = estimated covariance between random effects of the genome and those of the imputed transcriptome; and $\widehat{\sigma_\varepsilon^2}$ = estimated residual variance. The genetic architecture of the simulated trait is parameterised by linkage disequilibrium score, *w*, and minor allele frequency, *f*, in forms of $\mathrm{var}(\beta_i) \propto w_i^\gamma [f_i(1-f_i)]^{1+\alpha}$ for any given causal SNP *i*, where *α* and *γ* control the extents to which w and *f* influence the variance of SNP-specific effects on phenotypes, i.e., var(*β*), respectively. Panels

differ in the combination of α and γ values, which gives rise to different genetic architectures of the simulated trait. Under each genetic architecture, there are three scenarios for the covariance between the random effects of the genome and those of the transcriptome, i.e., cov=0, cov>0 and cov<0. For each scenario, 500 replicates of phenotypes (each with n = 10,000) were simulated; the wrong estimation model always assumes α = -1 and γ = 0, while the true model assumes the values of α and γ identical to those of the simulation model. Regardless of the estimation model, CORE GREML was applied for parameter estimation. Source data are provided as a Source Data file.

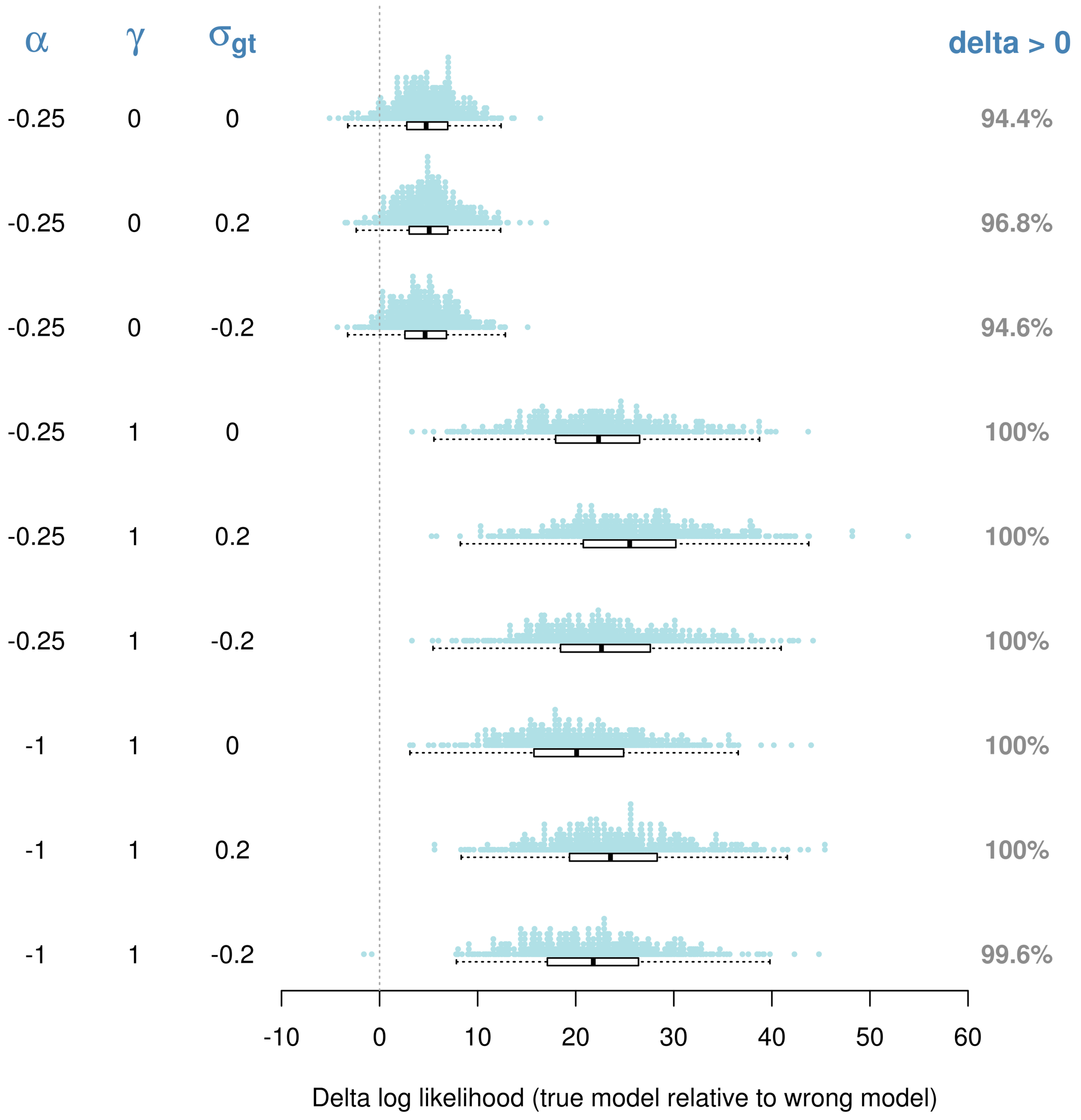

Supplementary Figure 5. Estimation model assuming the true genetic architecture of the simulated trait consistently shows a better fit than the model assuming a wrong genetic architecture. Shown in each row is the distribution of differences in log likelihood between the estimation model (true model) that assumes the correct genetic architecture of the simulated trait and a 'wrong model' that mis-specifies the genetic architecture. Irrespective of the estimation model, CORE GREML was used for parameter estimation. The difference in log likelihood is computed by subtracting the log likelihood of the wrong model from that of the true model, such that values above zero indicate that the true model has a better fit than the wrong model. The genetic architecture of the simulated trait is parameterised by linkage disequilibrium structure, *w*, and minor allele frequency, *f*, in forms of $\mathrm{var}(\beta_i) \propto w_i^{\gamma}[f_i(1-f_i)]^{1+\alpha}$ for any given causal SNP *i*, where α and γ control the extents to which *w* and *f* influence the variance of the SNP-specific effects on phenotypes, i.e., var(β), respectively. Combinations of α and γ values give rise to different genetic architectures (information displayed in columns 1 & 2 on the left). Under each genetic architecture, there are three scenarios for the covariance between the random effects of the genome and those of the transcriptome ($\sigma_{\mathrm{g\,t}}$; column 3 on the left). For each scenario, 500 replicates of phenotypes (each with n = 10,000) were simulated; the wrong estimation model always assumes α = -1 and γ = 0, while the true model assumes the values of α and γ identical to those of the simulation model. Box-plots elements: center line, median; box limits, upper and lower quartiles; whiskers, 1.5 x interquartile range. Source data are provided as a Source Data file.

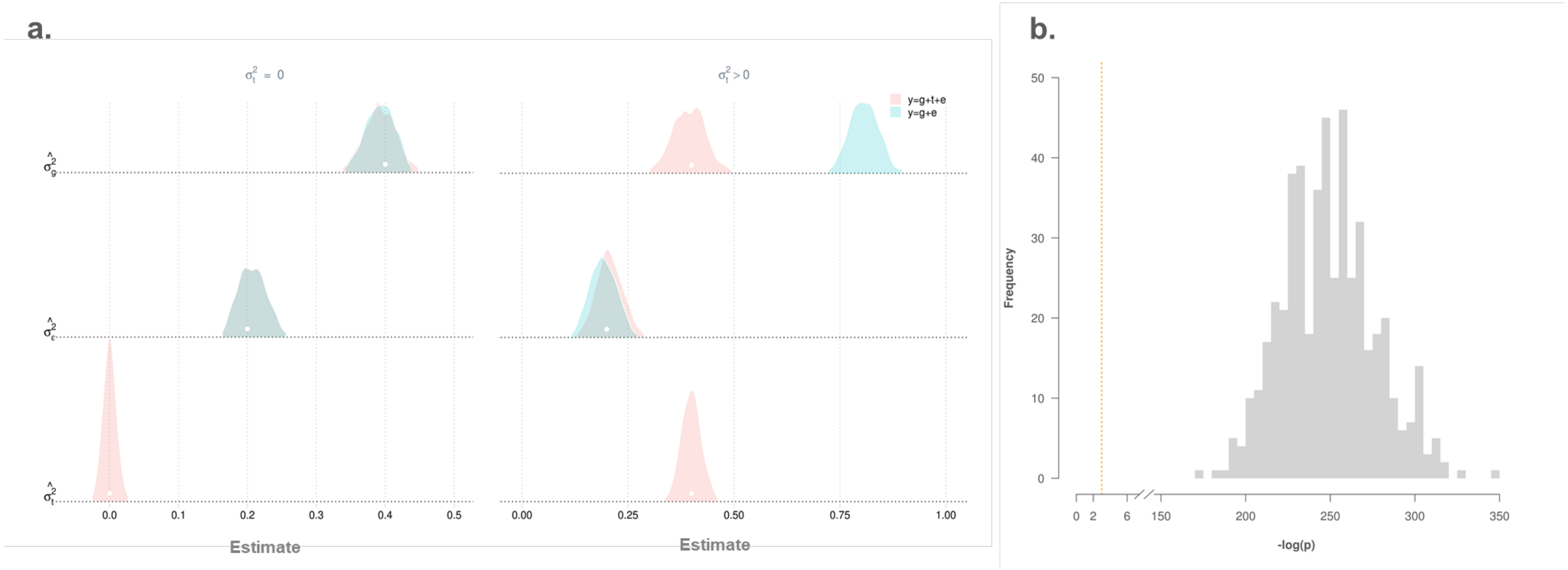

Supplementary Figure 6. GREML estimates of model parameters by estimation model and p-values for model comparisons. Five-hundred replicates of phenotypic data (n = 10,000) were simulated under settings with and without the random effects of the imputed transcriptome, denoted as $\sigma_t^2 > 0$ and $\sigma_t^2 = 0$, respectively. For each replicate, two linear mixed-effects models were fitted, one that did not include a term for random effects of the imputed transcriptome, i.e., **y** = **g** + **ε**, and the other did, i.e., **y** = **g** + **t** + **ε**. All model parameters were estimated using GREML. **Panel a**. estimated density of model parameters. $\widehat{\sigma_g^2}$ = estimated phenotypic variance explained by the genome; $\widehat{\sigma_t^2}$ = estimated phenotypic variance explained by the imputed transcriptome; and $\widehat{\sigma_\varepsilon^2}$ = estimated residual variance. True values are in dots. **Panel b**. histogram of p-values from likelihood ratio tests (df=1) that compared the two models (i.e., **y** = **g** + **ε** & **y** = **g** + **t** + **ε**.) for replicates simulated under $\sigma_t^2 > 0$. P-values are log transformed with the statistical significance threshold, i.e., -log (0.05), being indicated by the orange vertical dash line. Values above the threshold indicate that **y** = **g** + **t** + **ε** had a better fit than **y** = **g** + **ε**. Source data are provided as a Source Data file.

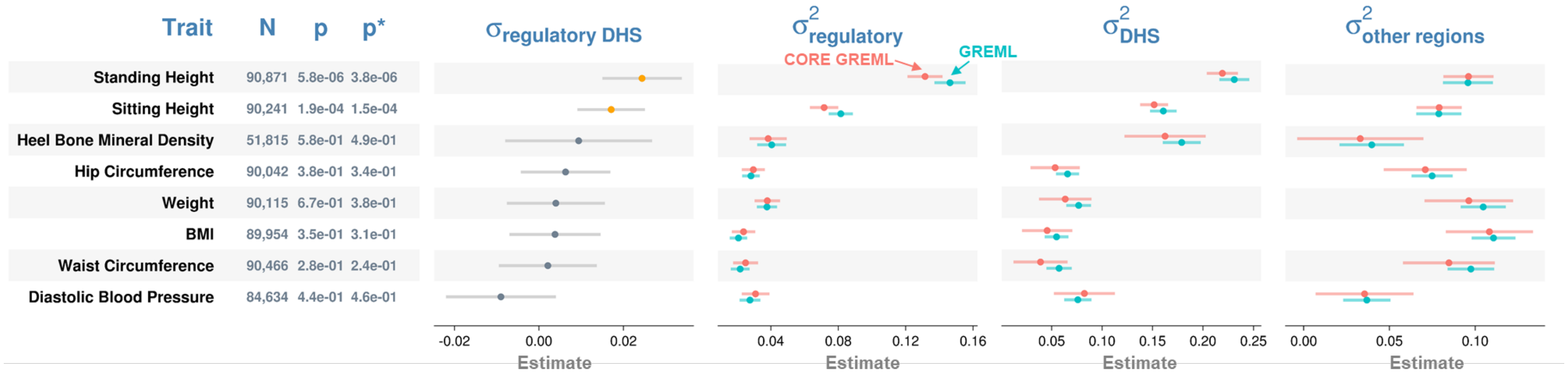

Supplementary Figure 7. Variance component estimates from genomic partitioning analyses. The three functional regions of the genome are regulatory regions, DNase I hypersensitivity sites (DHS), and all other regions. $\sigma^2_{\text{regulatory}}$, σ^2_{DHS} , and $\sigma^2_{\text{other regions}}$ denote phenotypic variances explained by the three functional regions; and $\sigma_{\text{regulatory DHS}}$ denotes the covariance between random effects of the regulatory regions and of the DHS on phenotypes. Error bars are 95% confidence intervals (based on s.e.m). Model parameters were estimated using the traditional method, i.e., GREML, and the proposed method, i.e., CORE GREML. N = sample size; p = p-values from likelihood ratio tests that compared GREML with CORE GREML to detect $\sigma_{\text{regulatory DHS}}$; and p* = p-values from a sensitivity analysis where a rank-based inverse normal transformation was applied to phenotypic data to check the robustness of signals against the violation of the normality assumption held by both GREML and CORE GREML. Highlighted in orange are significant $\sigma_{\text{regulatory DHS}}$ after a Bonferroni adjustment for multiple comparisons. Residual variance estimates are omitted for simplicity. Fluid intelligence and years of education are excluded because either the random effects of the regulatory regions or those of the DHS were not significant. Source data are provided as a Source Data file.

Trait	N	p	p*
Standing Height	90,871	5.3e-20	4.5e-20
Sitting Height	90,241	4.6e-15	3.5e-15
Heel Bone Mineral Density	51,815	1.5e-05	2.3e-05
Diastolic Blood Pressure	84,634	3.3e-04	3.4e-04
Hip Circumference	90,042	3.0e-02	2.6e-02
Weight	90,115	4.1e-01	2.5e-01
Waist Circumference	90,466	4.2e-01	3.7e-01
BMI	89,954	6.7e-01	5.6e-01
Years of Eduction	90,557	3.2e-01	2.6e-01

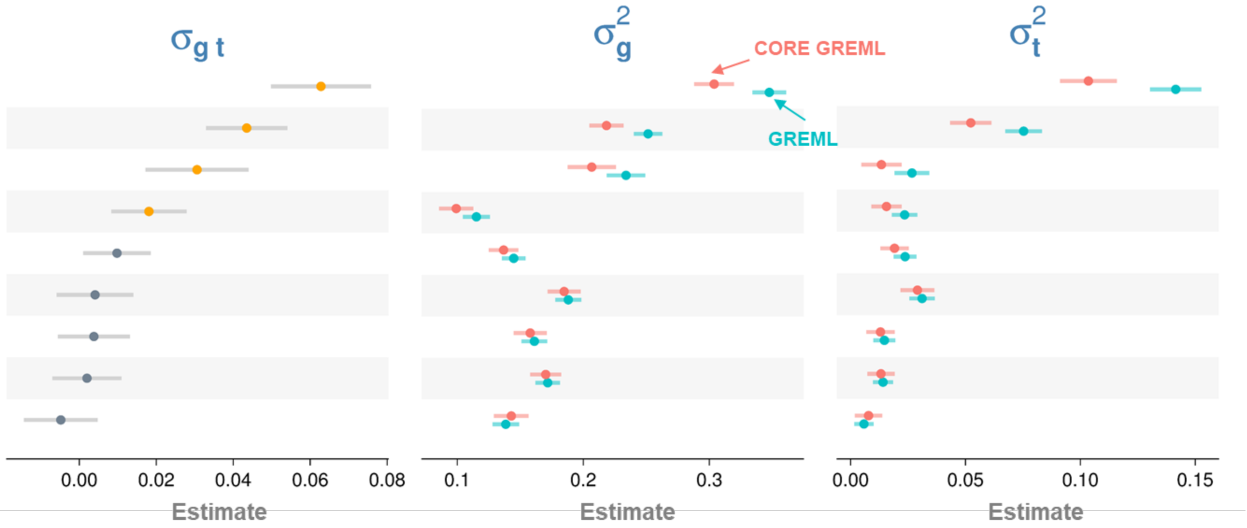

Supplementary Figure 8. Variance component estimates from genome-transcriptome partitioning analyses. σ_{g}^2 and σ_{t}^2 denote the phenotypic variances explained by the genome and by the imputed transcriptome, respectively; and $\sigma_{\mathrm{g\,t}}$ denotes the covariance between genetic effects and effects of imputed gene expressions on phenotypes. Model parameters were estimated using the traditional method, i.e., GREML, and the proposed method, i.e., CORE GREML. Error bars are 95% confidence intervals (based on s.e.m). N = sample size; p = p-values from likelihood ratio tests that compared GREML with CORE GREML to detect $\sigma_{\mathrm{g\,t}}$; and p* = p-values from a sensitivity analysis where a rank-based inverse normal transformation was applied to phenotypic data to check the robustness of signals against the violation of the normality assumption held by both GREML and CORE GREML. Highlighted in orange are significant $\sigma_{\mathrm{g\,t}}$ after a Bonferroni adjustment for multiple comparisons. Residual variance estimates are omitted for simplicity. Fluid intelligence is excluded because the random effects of the imputed transcriptome on this trait was not significant after Bonferroni correction. Source data are provided as a Source Data file.

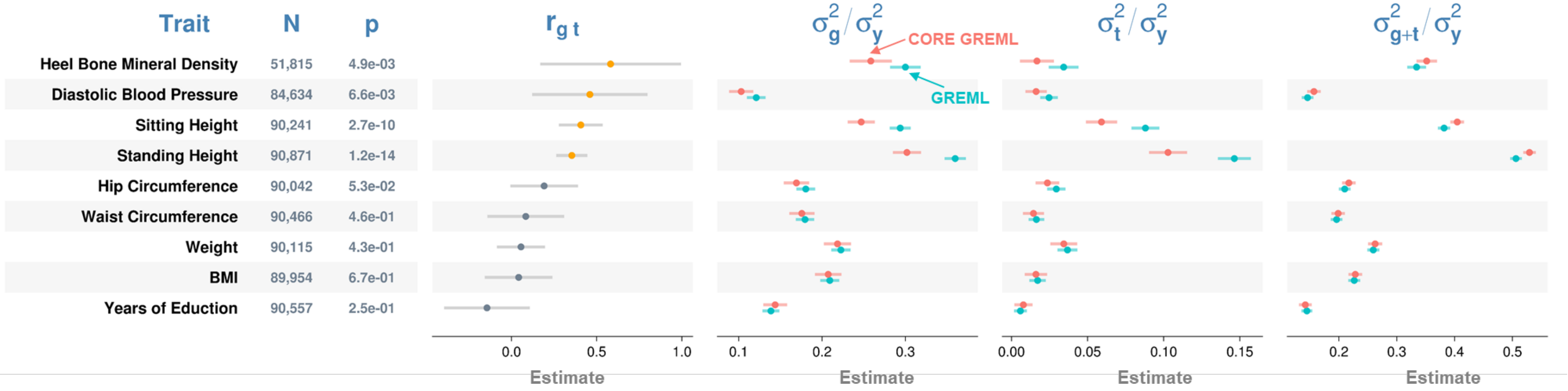

Supplementary Figure 9. Estimated proportions of phenotypic variance due to the genome and the imputed transcriptome. σ_g^2 and σ_t^2 denote the phenotypic variances explained by the genome and by the imputed transcriptome, respectively; $\sigma_{g+t}^2 = \sigma_g^2 + \sigma_t^2$; and $r_{g\,t}$ = correlation between the random effects of the genome and those of the imputed transcriptome. Model parameters were estimated using the traditional method, i.e., GREML, and the proposed method, i.e., CORE GREML. Error bars are 95% confidence intervals (based on s.e.m). N = sample size, and p = p-values are based on the Wald test statistic under the null hypothesis that $r_{g\,t} = 0$ (i.e., a two-sided test). Highlighted in orange are significant correlations after a Bonferroni adjustment for multiple comparisons. Fluid intelligence is excluded because the random effects of the imputed transcriptome for this trait were not significant after Bonferroni correction. Source data are provided as a Source Data file.

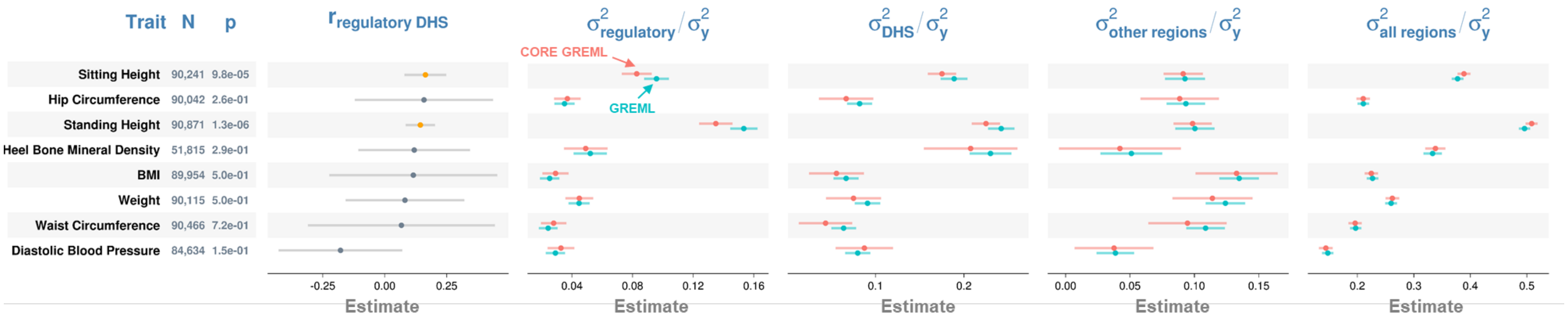

Supplementary Figure 10. Estimated proportions of phenotypic variance attributable to three functional genomic regions. The three functional regions are the regulatory regions, DNase I hypersensitivity sites (DHS), and all other regions. $\sigma^2_{\text{regulatory}}$, σ^2_{DHS} , and $\sigma^2_{\text{other regions}}$ denote phenotypic variances explained by the three functional regions; $\sigma^2_{\text{all regions}} = \sigma^2_{\text{regulatory}} + \sigma^2_{\text{DHS}} + \sigma^2_{\text{other regions}}$; σ^2_{y} = total phenotypic variance; and $r_{\text{regulatory DHS}}$ = correlation between the random effects of the regulatory region and DHS. Model parameters were estimated using the traditional method, i.e., GREML, and the proposed method, i.e., CORE GREML. Error bars are 95% confidence intervals (based on s.e.m). N = sample size, and p = p-values are based on the Wald test statistic under the null hypothesis that $r_{\text{regulatory DHS}} = 0$ (i.e., a two-sided test). Highlighted in orange are significant correlations after a Bonferroni adjustment for multiple comparisons. Fluid intelligence and years of education are excluded because either the random effects of the regulatory regions or those of the DHS were not significant. Source data are provided as a Source Data file.

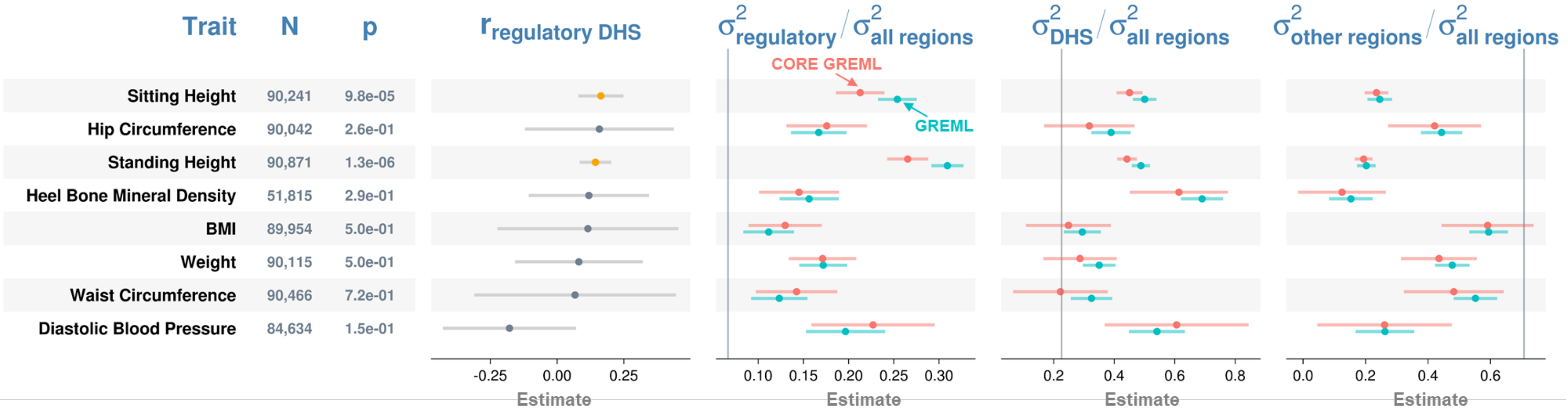

Supplementary Figure 11. Estimated proportions of total genetic variance attributable to three functional regions of the genome. The three functional regions are the regulatory regions, DNase I hypersensitivity sites (DHS), and all other regions. $\sigma^2_{\text{regulatory}}$, σ^2_{DHS} , and $\sigma^2_{\text{other regions}}$ denote phenotypic variances explained by the three functional regions. $\sigma^2_{\text{all regions}}$= total genetic variance of all three functional regions; and $r_{\text{regulatory DHS}}$ = correlation between the random effects of the regulatory regions and DHS. Model parameters were estimated using the traditional method, i.e., GREML, and the proposed method, i.e., CORE GREML. Error bars are 95% confidence intervals (based on s.e.m). N = sample size, and p = p-values based on the Wald test statistic under the null hypothesis that $r_{\text{regulatory DHS}}$ = 0 (i.e., a two-sided test). Highlighted in orange are significant correlations after a Bonferroni adjustment for multiple comparisons. Vertical lines are percentages of SNPs from the three functional regions, and conceptually, they are expected proportions of total genetic variance explained by the three functional regions of the genome assuming all genome-wide SNPs have an equal contribution to phenotypic variation. Fluid intelligence and years of education are excluded because either the random effects of the regulatory regions or those of the DHS were not significant. Source data are provided as a Source Data file.

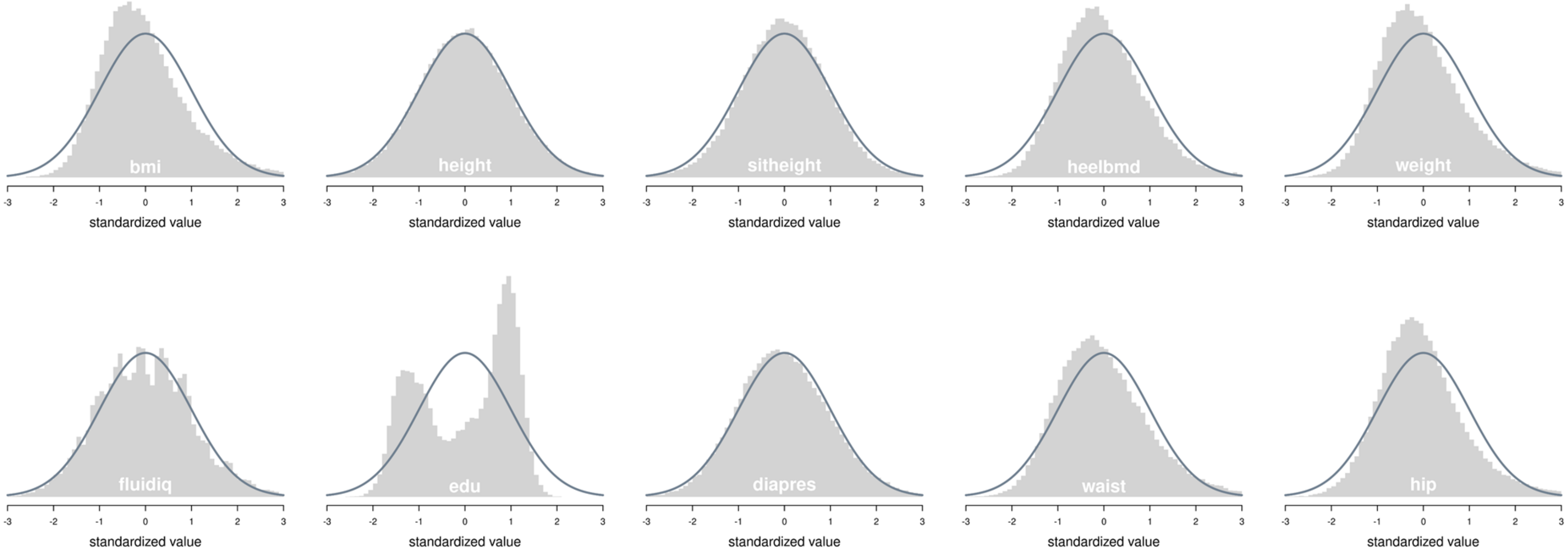

Supplementary Figure 12. Histograms of phenotypic data for ten selected traits from the UK Biobank. Traits from top left to bottom right are body mass index, standing height, sitting height, heel bone mineral density, weight, fluid intelligence, years of education, diastolic blood pressure, waist circumference and hip circumference. Data were prepared in three sequential steps: 1) adjustment for age, sex, birth year, social economic status, population structure, assessment centre, and genotype batch; 2) standardization; and 3) removal of data points outside +/- 3 standard deviations from the mean. The density function of Normal (0, 1) is superimposed as the reference to highlight deviations from normality.

Supplementary Table 1. True model parameter values for simulation models under three parameter settings.

Simulation Model	Model Parameter	Parameter Setting		
		cov=0	*cov>0*	*cov<0*
**genome-transcriptome model**	σ^2_g	0.4	0.4	0.4
$\mathbf{y} = \mathbf{g} + \mathbf{t} + \boldsymbol{\varepsilon}$, where	σ^2_t	0.4	0.4	0.4
$\mathbf{g} \sim N(\mathbf{0}, \mathbf{A}\sigma^2_g)$, $\mathbf{t} \sim N(\mathbf{0}, \mathbf{T}\sigma^2_t)$ & $\boldsymbol{\varepsilon} \sim N(\mathbf{0}, \mathbf{I}\sigma^2_\varepsilon)$	σ^2_ε	0.2	0.2	0.2
	σ_{gt}	0	0.2	-0.2
**genomic partitioning model**	$\sigma^2_{regulatory}$	0.3	0.3	0.3
$\mathbf{y} = \mathbf{g}_{regulatory} + \mathbf{g}_{DHS} + \mathbf{g}_{other\ regions} + \boldsymbol{\varepsilon}$	σ^2_{DHS}	0.4	0.4	0.4
where $\mathbf{g_i} \sim N(\mathbf{0}, \mathbf{A_i}\sigma^2_i)$ for $i \in \{regulatory, DHS, other\ regions\}$	$\sigma^2_{other\ regions}$	0.1	0.1	0.1
& $\boldsymbol{\varepsilon} \sim N(\mathbf{0}, \mathbf{I}\sigma^2_\varepsilon)$	σ^2_ε	0.2	0.2	0.2
	$\sigma_{regulatory\,DHS}$	0	0.2	-0.2
	$\sigma_{regulatory\,other\ regions}$	0	0.1	-0.1
	$\sigma_{DHS\,other\ regions}$	0	0.05	-0.05

A & **T** are kernel matrices constructed using genotypes of 1,131,002 SNPs and using imputed expression levels of 227,664 genes collapsed across 43 tissues, respectively. **I** is an identity matrix. $\mathbf{A}_i$ is the genomic relationship matrix constructed using SNPs from functional region *i* of the genome.

Supplementary Table 2. The number of genes for which expression levels were imputed across 43 non-sex-specific tissues.

Tissue	No. of Genes
Adipose Subcutaneous	8,244
Adipose Visceral Omentum	6,571
Adrenal Gland	4,590
Artery Aorta	6,636
Artery Coronary	3,486
Artery Tibial	8,217
Brain Amygdala	2,349
Brain Anterior cingulate cortex BA24	3,291
Brain Caudate basal ganglia	4,164
Brain Cerebellar Hemisphere	4,743
Brain Cerebellum	6,075
Brain Cortex	4,321
Brain Frontal Cortex BA9	3,583
Brain Hippocampus	2,806
Brain Hypothalamus	2,826
Brain Nucleus accumbens basal ganglia	3,621
Brain Putamen basal ganglia	3,161
Brain Spinal cord cervical c-1	2,508
Brain Substantia nigra	2,028
Breast Mammary Tissue	5,291
Cells EBV-transformed lymphocytes	3,027
Cells Transformed fibroblasts	7,335
Colon Sigmoid	5,220
Colon Transverse	5,587
Esophagus Gastroesophageal Junction	5,240
Esophagus Mucosa	8,153
Esophagus Muscularis	7,720
Heart Atrial Appendage	5,964
Heart Left Ventricle	5,354
Liver	3,336
Lung	7,938
Minor Salivary Gland	2,217
Muscle Skeletal	7,506
Nerve Tibial	9,414
Pancreas	5,320
Pituitary	4,752
Skin Not Sun Exposed Suprapubic	7,621
Skin Sun Exposed Lower leg	9,093
Small Intestine Terminal Ileum	3,093
Spleen	4,580
Stomach	4,776
Thyroid	9,630
Whole Blood	6,277

Note: transcriptome imputation was based on GTEx V7 models (http://predictdb.org/).

Supplementary Table 3. Heritability estimates by estimation model.

Trait	GCTA model*			LDAK model*			GCTA vs. LDAK
	h2	se	log likelihood	h2	se	log likelihood	Δ log likelihood**
BMI	0.23	5.10E-03	-34638	0.23	5.80E-03	-35123	485
Standing Height	0.50	5.10E-03	-37071	0.51	5.40E-03	-38723	1652
Sitting Height	0.38	5.30E-03	-34158	0.39	5.80E-03	-35310	1152
Heel Bone Mineral Density	0.33	8.20E-03	-18322	0.35	9.50E-03	-18711	389
Weight	0.26	5.20E-03	-35553	0.27	5.90E-03	-36147	594
Fluid Intelligence	0.23	1.26E-02	-14414	0.26	1.59E-02	-14480	66
Years of Education	0.14	4.70E-03	-44585	0.16	5.60E-03	-44807	221
Diastolic Blood Pressure	0.15	5.10E-03	-39583	0.15	6.00E-03	-39816	233
Waist Circumference	0.20	5.00E-03	-39197	0.20	5.80E-03	-39594	398
Hip Circumference	0.21	5.10E-03	-33817	0.22	5.80E-03	-34246	429

*Fitted GCTA model and LDAK model differ in the assumption about the variance of SNP-specific effects on phenotypes, $\mathrm{var}(\beta)$. In general, it is assumed that for a given SNP *i*, the variance of its effects on phenotypes is proportional to its linkage disequilibrium score, *w*, and minor allele frequency, *f*, i.e.,$\mathrm{var}(\beta_i) \propto w_i^{\gamma}[f_i(1-f_i)]^{1+\alpha}$, where parameter γ modulates the effect of *w* and α modulates the effect of *f* on $\mathrm{var}(\beta_i)$. In the GCTA model, α and γ were assumed to be -1 and 0, respectively; in the LDAK model, γ was assumed to 1 and α was set to the recommended default, -0.25[1]. Regardless of the estimation model, GREML was used for heritability estimation. **Δ log likelihood was derived by subtracting the log likelihood of the LDAK model from that of the GCTA model.

Supplementary Table 4. Likelihood of estimation models when GREML and CORE GREML were applied for the genome-transcriptome partitioning of phenotypic variance.

	log likelihood of GCTA* model			log likelihood of LDAK* model			GCTA vs. LDAK
**Trait**	GREML	CORE GREML	*p-value****	GREML	CORE GREML	*p-value*	Δ log likelihood (CORE GREML)**
BMI	-34611	-34611	6.70E-01	-34956	-34950	4.67E-04	339
Standing Height	-36423	-36381	5.31E-20	-37024	-36983	5.64E-20	601
Sitting Height	-33829	-33799	4.64E-15	-34330	-34297	6.67E-16	498
Heel Bone Mineral Density	-18292	-18283	1.53E-05	-18535	-18525	5.77E-06	242
Weight	-35458	-35458	4.13E-01	-35818	-35811	2.39E-04	353
Fluid Intelligence	-14411	-14409	2.10E-02	-14459	-14458	1.37E-01	49
Years of Education	-44581	-44580	3.21E-01	-44756	-44754	3.91E-02	174
Diastolic Blood Pressure	-39530	-39523	3.34E-04	-39648	-39640	1.22E-04	117
Waist Circumference	-39170	-39169	4.25E-01	-39451	-39445	3.42E-04	275
Hip Circumference	-33746	-33744	2.97E-02	-34003	-33992	2.42E-06	248

*Fitted GCTA model and LDAK model differ in the assumption about the variance of SNP-specific effects on phenotypes, $\mathrm{var}(\beta)$. In general, it is assumed that for a given SNP *i*, the variance of its effects on phenotypes is proportional to its linkage disequilibrium score, *w*, and minor allele frequency, *f*, i.e.,$\mathrm{var}(\beta_i) \propto w_i^{\gamma}[f_i(1-f_i)]^{1+\alpha}$, where parameter *γ* modulates the effect of *w* and *α* modulates the effect of *f* on $\mathrm{var}(\beta_i)$. In the GCTA model, *α* and *γ* were assumed to be -1 and 0, respectively; in the LDAK model, *γ* was assumed to 1 and *α* was set to the recommended default, -0.25[1]. **Δ log likelihood was derived by subtracting the log likelihood of the LDAK model from that of the GCTA model, when CORE GREML was used for parameter estimation for both models.***p-values are from likelihood ratio tests that compared GREML with CORE GREML to detect the covariance between random effects of the genome and those of the transcriptome on phenotypes. P-values are not adjusted for multiple comparisons.

Supplementary Table 5. GREML estimates of genetic variance partitioned by functional genomic region.

Trait	Regulatory Regions		DHS		Other Regions	
	est. (s.e.)	*p*	*est. (s.e.)*	*p*	*est. (s.e.)*	*p*
BMI	0.02 (2.6E-03)	1.20E-15	0.05 (5.9E-03)	1.30E-20	0.11 (6.4E-03)	5.00E-67
Weight	0.04 (3.0E-03)	3.20E-36	0.08 (6.1E-03)	2.40E-36	0.1 (6.6E-03)	1.10E-56
Fluid Intelligence	0.02 (7.6E-03)	1.20E-02	0.07 (1.8E-02)	8.20E-05	0.14 (1.9E-02)	6.50E-13
Years of Eduction	0.01 (2.8E-03)	2.20E-02	0.03 (6.5E-03)	2.90E-05	0.11 (7.2E-03)	3.00E-53
Waist Circumference	0.02 (2.8E-03)	6.90E-15	0.06 (6.3E-03)	8.20E-20	0.1 (6.8E-03)	1.30E-46
Hip Circumference	0.03 (2.6E-03)	2.10E-27	0.07 (5.7E-03)	7.90E-31	0.07 (6.0E-03)	9.20E-36
Standing Height	0.15 (4.6E-03)	5.60E-222	0.23 (7.4E-03)	6.40E-214	0.1 (7.3E-03)	2.40E-39
Sitting Height	0.08 (3.6E-03)	1.80E-113	0.16 (6.6E-03)	6.00E-131	0.08 (6.6E-03)	7.40E-33
Heel Bone Mineral Density	0.04 (4.3E-03)	4.60E-21	0.18 (9.4E-03)	9.30E-81	0.04 (9.4E-03)	2.40E-05
Diastolic Blood Pressure	0.03 (3.1E-03)	5.40E-19	0.08 (6.8E-03)	5.30E-29	0.04 (6.9E-03)	9.60E-08

Note: p-values are based on the Wald test statistic with one degree of freedom under the null hypothesis that the variance component of interest is zero (i.e., a two-sided test). P-values are not adjusted for multiple comparisons.

Supplementary Table 6. The effects of the imputed transcriptome on phenotypes are orthogonal to the effects of SNPs used for the transcriptome imputation.

	(a) $y = g_1 + t + \varepsilon$ VS. (b) $y = g_1 + \varepsilon$				(c) $y = g_0 + g_1 + \varepsilon$ VS. (d) $y = g_0 + t + \varepsilon$		
**Trait**	log-likelihood $_a$	log-likelihood $_b$	Δ log-likelihood $_{a-b}$	p-value	log-likelihood $_c$	log-likelihood $_d$	Δ log-likelihood $_{d-c}$
Standing Height	-36627	-36968	342	1.19E-150	-36754	-36423	330
BMI	-34875	-34891	16	9.97E-09	-34635	-34611	24
Sitting Height	-33948	-34096	149	1.03E-66	-33950	-33829	121
Weight	-35720	-35776	56	2.39E-26	-35531	-35458	73
Years of Education	-44748	-44751	2	2.70E-02	-44585	-44581	4
Diastolic Blood Pressure	-39584	-39611	27	1.83E-13	-39558	-39530	28
Waist Circumference	-39376	-39394	17	5.21E-09	-39194	-39170	24
Hip Circumference	-33907	-33950	43	2.45E-20	-33798	-33746	52
Heel Bone Mineral Density	-18322	-18331	9	1.65E-05	-18278	-18292	-15
Fluid Intelligence	-14445	-14445	0	5.46E-01	-14412	-14411	1

Notes. 1. **y** = phenotypes; **t** = random effects of the imputed transcriptome; $\mathbf{g_0}$ = random effects of 1,074,458 SNPs for genome-transcriptome partitioning analyses presented throughout the main text; $\mathbf{g_1}$ = random effects of 1,316,391 SNPs used for the transcriptome imputation; **ε** = residuals. Log-likelihood$_x$ = log-likelihood of model x, where x = a, b, c, or d; Δ log-likelihood$_{x\text{-}y}$ = log-likelihood of model x – log-likelihood of model y, where x = a or b and y = c or d. 2; and p-values are from likelihood ratio tests that compared models a and b. P-values are not adjusted for multiple comparisons. It is important to distinguish the SNP sets involved for different variance components of the models. The kernel matrix for **t** is based on gene expressions imputed using 1,316,391 SNPs, which is the same set of SNPs used to construct the kernel matrix for $\mathbf{g_1}$. This set of SNPs only has ~ 600K in common with the 1,074,458 SNPs used to construct the kernel matrix for $\mathbf{g_0}$. In our primary analysis (model a VS. model b), we show that model a has a better fit than model b for eight traits, even though **t** and $\mathbf{g_1}$ are based on the same set of SNPs. Hence, **t** is orthogonal to $\mathbf{g_1}$ for most traits. In our secondary analysis (model c VS. model d), we show that model d has a better fit than model c for seven traits—once again—despite the fact that **t** and $\mathbf{g_1}$ are based on the same set of SNPs. This confirms that **t** and $\mathbf{g_1}$ are distinct for most traits.

Supplementary Table 7. Genome-Transcriptome covariance estimates based on HapMap phase II and HapMap phase III SNPs.

Trait	N	$\sigma_{g0\ t}$			$\sigma_{g1\ t}$		
		est	*se*	*p*	*est*	*se*	*p*
BMI	89,954	0.002	0.005	6.70E-01	-0.005	0.005	3.33E-01
Weight	90,115	0.004	0.005	4.13E-01	0.000	0.006	9.40E-01
Fluid Intelligence	29,707	0.028	0.012	2.10E-02	0.042	0.014	3.23E-03
Years of Education	90,557	-0.005	0.005	3.21E-01	-0.004	0.005	5.16E-01
Waist Circumference	90,466	0.004	0.005	4.25E-01	0.000	0.005	9.68E-01
Hip Circumference	90,042	0.010	0.004	2.97E-02	0.004	0.005	4.11E-01
Standing Height	90,871	0.063	0.007	5.31E-20	0.051	0.008	1.16E-10
Sitting Height	90,241	0.044	0.005	4.64E-15	0.038	0.006	6.71E-09
Heel Bone Mineral Density	51,815	0.031	0.007	1.53E-05	0.011	0.008	1.64E-01
Diastolic Blood Pressure	84,634	0.018	0.005	3.34E-04	0.014	0.006	1.36E-02

Note: p-values are from likelihood ratio tests with one degree of freedom that compared GREML with CORE GREML to detect the genome-transcriptome covariance term, i.e., $\sigma_{g0\ t}$ or $\sigma_{g1\ t}$. P-values are not adjusted for multiple comparisons. The kernel matrix for $\mathbf{g_1}$ is based on 1,316,391 HapMap phase II SNPs, and the kernel matrix for $\mathbf{g_0}$ is based on 1,074,458 HapMap phase III SNPs, which has ~600K SNPs in common with the HapMap phase II SNPs for $\mathbf{g_1}$. The kernel matrix for **t** is based on gene expressions imputed using the same set of 1,316,391 HapMap phase II SNPs as for $\mathbf{g_1}$.

Supplementary Table 8. Correlations between off-diagonal entries of kernel matrices used for variance-components estimation.

	$\mathbf{A}_{regulatory}$	$\mathbf{A}_{DHS}$	$\mathbf{A}_{other}$	**A**	**T**
$\mathbf{A}_{regulatory}$	1				
$\mathbf{A}_{DHS}$	0.46	1			
$\mathbf{A}_{other}$	0.46	0.78	1		
**A**	0.57	0.87	0.98	1	
**T**	0.54	0.37	0.35	0.42	1

A, $\mathbf{A}_{regulatory}$, $\mathbf{A}_{DHS}$ and $\mathbf{A}_{other}$ are genomic relationship matrices constructed using available SNPs from the entire genome, the functional regions, the DHS and all other regions, respectively. **T** is a kernel matrix constructed using the imputed expression levels of genes collapses across 43 tissues. Note all matrices are based on data from 10,000 UK biobank participants randomly selected for our simulations.

Supplementary Table 9. Computational resource requirements of CORE GREML for fitting a model with two random effects.

sample size	RAM (GB)	duration (h)	
		reading	*computing*
90,000	161	7.0	29.2
70,000	97	2.5	13.7
50,000	51	1.2	5.2
30,000	19	0.4	1.1
10,000	3	0.05	0.1

Note: All analyse were performed using mtg2.14, with each running on a single central processing unit (CPU) at a speed between 1200 and 3200 MHz. Task duration is partitioned into time taken for reading in kernel matrices and computing time. RAM = random access memory. When using multiple CPUs with parallel computing, the computing time is expected to reduce in a curvilinear fashion. Since CORE GREML uses the direct AI algorithm [2,3], the computational efficiency is robust to increasing number of random effects including their covariance terms in the model.

Supplementary Note 1

In this note, we report how well GREML can capture the effects of the imputed transcriptome, noting that GREML does not explicitly model the effects of the imputed transcriptome. We simulated 500 replicates of phenotypic data using the genome-transcriptome model (Supplementary Table 1) under settings with and without random effects of the imputed transcriptome (σ_{t}^2 = 0 vs. σ_{t}^2 = 0.4). For both settings, the covariance between random effects of the genome and those of the imputed transcriptome was set to zero. For each replicate, we fitted two models, a 'G model' that breaks phenotypic effects into random effects of the genome and residuals, i.e., $\mathbf{y} = \mathbf{g} + \boldsymbol{\varepsilon}$, and a 'G-T model' that decomposes phenotypic effects into random effects of genome and of the imputed transcriptome and residuals, i.e., $\mathbf{y} = \mathbf{g} + \mathbf{t} + \boldsymbol{\varepsilon}$; both models were estimated using GREML. We declared the presence of transcriptomic effects when the G-T model had a better fit than the G model via a likelihood ratio test with one degree of freedom. We found that the type I error rate was controlled (0.042) under the zero transcriptomic effects setting. Under both settings, G-T model yielded unbiased estimates of model parameters (Supplementary Figure 6a). On the other hand, the G model produced unbiased estimates only under the null setting, as expected. Interestingly, in the presence of transcriptomic effects, the estimated genetic variance by the G model was equivalent to the sum of estimated variances due to the genome and imputed transcriptome by the G-T model (Supplementary Figure 6a), such that the phenotypic variance explained by the two models were similar. This indicates that SNP-based heritability estimates by GREML can be inflated by phenotypic variance due to the transcriptome. Importantly, although both models captured a similar amount of phenotypic variance, the fit of the G-T model was far better than that of the G model (Supplementary Figure 6b), indicating the partition of phenotypic variance represented by the G-T model is closer to the truth than that represented by the G model.

Supplementary References

1. Speed, D., Cai, N., Johnson, M. R., Nejentsev, S., Balding, D. J. & Consortium, U. Reevaluation of SNP heritability in complex human traits. *Nat. Genet.* **49**, 986-992 (2017).

2. Lee, S. H., Van Der Werf & J. H. An efficient variance component approach implementing an average information REML suitable for combined LD and linkage mapping with a general complex pedigree. *Genet Sel. Evol.* **38**, 25-43 (2006).

3. Yang, J., Lee, S. H., Goddard, M. E. & Visscher, P. M. GCTA: a tool for genome-wide complex trait analysis. *Am. J. Hum. Genet.* **88**, 76-82 (2011).
