# Supplementary material for: CORE GREML for estimating covariance between random effects in linear mixed models for complex trait analyses
Source: Nat Commun. 2020 Aug 21;11:4208. doi: 10.1038/s41467-020-18085-5 (PMC7442840; doi:10.1038/s41467-020-18085-5)
Supplement: Supplementary file 3 — Reporting Summary [file 41467_2020_18085_MOESM3_ESM.pdf]

nature research

Corresponding author(s): S. Hong Lee

Last updated by author(s): 23/07/2020

Reporting Summary

Nature Research wishes to improve the reproducibility of the work that we publish. This form provides structure for consistency and transparency in reporting. For further information on Nature Research policies, see Authors & Referees and the Editorial Policy Checklist.

Statistics

For all statistical analyses, confirm that the following items are present in the figure legend, table legend, main text, or Methods section.

n/a	Confirmed	
☐	☒	The exact sample size (n) for each experimental group/condition, given as a discrete number and unit of measurement
☐	☒	A statement on whether measurements were taken from distinct samples or whether the same sample was measured repeatedly
☐	☒	The statistical test(s) used AND whether they are one- or two-sided<br>*Only common tests should be described solely by name; describe more complex techniques in the Methods section.*
☐	☒	A description of all covariates tested
☐	☒	A description of any assumptions or corrections, such as tests of normality and adjustment for multiple comparisons
☐	☒	A full description of the statistical parameters including central tendency (e.g. means) or other basic estimates (e.g. regression coefficient) AND variation (e.g. standard deviation) or associated estimates of uncertainty (e.g. confidence intervals)
☐	☒	For null hypothesis testing, the test statistic (e.g. F, t, r) with confidence intervals, effect sizes, degrees of freedom and P value noted<br>*Give P values as exact values whenever suitable.*
☒	☐	For Bayesian analysis, information on the choice of priors and Markov chain Monte Carlo settings
☒	☐	For hierarchical and complex designs, identification of the appropriate level for tests and full reporting of outcomes
☐	☒	Estimates of effect sizes (e.g. Cohen's d, Pearson's r), indicating how they were calculated

Our web collection on statistics for biologists contains articles on many of the points above.

Software and code

Policy information about availability of computer code

Data collection	No software was used for data collection. The data used in this study were provided by the UK Biobank, and our access to the data was under the reference number 14575.
Data analysis	We used MTG2 (v2.14), R (v3.4.3), Plink (v1.9), PrediXcan to process and analze data in this study. Example code along with related files for fitting CORE GREML using MTG2 can be accessed without any restrictions from https://sites.google.com/site/honglee0707/mtg2

For manuscripts utilizing custom algorithms or software that are central to the research but not yet described in published literature, software must be made available to editors/reviewers. We strongly encourage code deposition in a community repository (e.g. GitHub). See the Nature Research guidelines for submitting code & software for further information.

Data

Policy information about availability of data

All manuscripts must include a data availability statement. This statement should provide the following information, where applicable:

- Accession codes, unique identifiers, or web links for publicly available datasets
- A list of figures that have associated raw data
- A description of any restrictions on data availability

The UK Biobank data can be accessed through procedures described on its webpage (http://www.ukbiobank.ac.uk/using-the-resource/). The source data underlying Figures 1-6 and Supplementary Figures 1-11 are provided in the source data file. Simulated data used in this paper can be obtained from the authors upon request.

Field-specific reporting

Please select the one below that is the best fit for your research. If you are not sure, read the appropriate sections before making your selection.

☒ Life sciences ☐ Behavioural & social sciences ☐ Ecological, evolutionary & environmental sciences

For a reference copy of the document with all sections, see nature.com/documents/nr-reporting-summary-flat.pdf

Life sciences study design

All studies must disclose on these points even when the disclosure is negative.

Sample size	We analyzed all available records of 10 traits from the UK Biobank, and the sample size varies across traits, ranging from 51,815 (heel bone mineral density) to 90,871 (height). Based on our simulations that use a sample size of 10,000 individuals, the estimated power of detecting a small to moderate correlation between random effects (i.e., r between 0.25 and 0.6) is 100%. Given that the sample size of real data is 5 to 9 times greater than that for our simulations, our analyses of real data are well-powered.
Data exclusions	We applied well established (hence widely used) quality control procedures to exclude unreliable genotypic data. The data exclusion was performed before fitting any of our models (i.e., genome-transcriptome partitioning model & genomic partitioning models). The rationale behind the data exclusion is to avoid bias in our results due to poor quality data. The quality control procedures are detailed as follows:<br><br>We filtered SNPs with an INFO score < 0.6, a MAF < 0.01, a Hardy-Weinberg equilibrium p-value <1e-4, or a call rate < 0.95. We then selected HapMap3 SNPs, which are known to yield reliable and robust estimates of SNP-based heritability, for downstream analyses. We excluded individuals who had a genotype-missing rate > 0.05, were non-white British ancestry, or had the first or second ancestry principal components outside six standard deviations of the population mean. We also applied relatedness-cut-off quality control to exclude one of any pair of individuals with a genomic relationship > 0.025. From the remaining individuals, we selected those who were included in both the first and second release of UK biobank genotype data. We calculated the discordance rate of imputed genotypes between the two versions and excluded individuals with a discordance rate > 0.05.
Replication	Using 5-fold cross-validation, we validated the transcriptomic effects on phenotypes and their correlations with genetic effects on phenotypes (see Figure 2 of the manuscript for results), indicating that these findings are replicable.
Randomization	For our 5-fold cross-validation, we randomly assigned participants to training and target sets.
Blinding	Blinding was not applicable to our study, because the data were collected by the UK Biobank and the ID of participants has been encrypted.

Reporting for specific materials, systems and methods

We require information from authors about some types of materials, experimental systems and methods used in many studies. Here, indicate whether each material, system or method listed is relevant to your study. If you are not sure if a list item applies to your research, read the appropriate section before selecting a response.

Materials & experimental systems

n/a	Involved in the study	
☒	☐	Antibodies
☒	☐	Eukaryotic cell lines
☒	☐	Palaeontology
☒	☐	Animals and other organisms
☐	☒	Human research participants
☒	☐	Clinical data

Methods

n/a	Involved in the study	
☒	☐	ChIP-seq
☒	☐	Flow cytometry
☒	☐	MRI-based neuroimaging

Human research participants

Policy information about studies involving human research participants

Population characteristics	UK Biobank data consist of 500,000 participants aged between 40-69 years in 2006-2010, with ~54% females and 46% males. Approximately 50,000 samples were genotyped on the UK BiLEVE Axiom array and the other 450,000 samples were genotyped on the UK Biobank Axiom array. Genotype were imputed to the whole genome level with IMPUTE3 using UK10K and 1000 Genome Phase 3 as the reference.
Recruitment	The recruitment includes a half million people from all around the UK who are currently aged 40-69 because the age group involves people at risk over the next few decades of developing a wide range of important diseases, conditions and covariates. The phenotype and genotype information is based on extensive baseline questionnaire and physical measures, as well as stored blood and urine samples that allow many different types of assay, incorporated with information from the UK National Health Service.

The UK Biobank may not be representative of the general population due to a low response rate and the "healthy volunteer" selection bias (PMID 28641372). However, the effects of bias appear to be subtle in a recent study (PMID 26849114). In fact, such bias is more problematic for a study without validation of estimated effects. However, in our study we explicitly validated the estimated transcriptomic effects and their correlations with genomic effects in the target set for all traits.

Ethics oversight

The UK Biobank's scientific protocol has been reviewed and approved by the North West Multi-centre Research Ethics Committee (MREC), National Information Governance Board for Health & Social Care (NIGB), and Community Health Index Advisory Group (CHIAG). UK Biobank has obtained informed consent from all participants. Our access to the UK Biobank data was under the reference number 14575. The research ethics approval of the current study was obtained from the University of South Australia Human Research Ethics Committee.

Note that full information on the approval of the study protocol must also be provided in the manuscript.
