# Supplementary material for: CORE GREML for estimating covariance between random effects in linear mixed models for complex trait analyses
Source: Nat Commun. 2020 Aug 21;11:4208. doi: 10.1038/s41467-020-18085-5 (PMC7442840; doi:10.1038/s41467-020-18085-5)
Supplement: Supplementary file 2 — Peer Review File [file 41467_2020_18085_MOESM2_ESM.docx]

Peer Review Information

**Journal:** Nature Communications

**Manuscript title:** CORE GREML for estimating covariance between random effects in linear mixed models for complex trait analyses

**Corresponding author name(s):** Dr Sang Hong Lee

**Editorial notes:**

**none**

**Reviewer comments & decisions:**

**Reviewer comments, first version:**

Reviewer #1 (Remarks to the Author):

Lee et al propose Core GREML, which generalizes GREML by including covariance terms to allow for correlations between random effects. I see importance in the proposed method, especially (as the authors suggest) for multiomic analyses - ie, deciding the relative contributions of different classes of genetic variation. For example, we know that SNPs explain substantial variance of phenotypes, and that gene expressions also explain substantial. However, because snps and gene expressions are correlated, it is hard to know to what extent snps explain variance because they influence gene expression, or to what extent gene expressions appear to explain variance because they correlate with SNPs.

Major comments

The method seems accurate (mathematically, adding covariances is close to trivial, so existing solvers can be used to estimate variance components). However, I consider the two applications quite poor.

1 - The first (as described above) seeks to estimate the relative contributions of SNPs and gene expressions to phenotypes. A major shortcoming of this analysis is that, because (actual) gene expression data are not available for UKBB, the authors instead use imputed gene expression. These are obtained by first constructing simple (generally 1, 2 or 3 parameter) SNP-prediction models for gene expressions from a reference transcription database. The predicted gene expression tend to explain a relatively small fraction of gene expression variation and thus the final estimate of variance explained by gene expression will likely dramatically underestimate the true contribution of gene expression (the authors acknowledge under-estimation, but I think do not appreciate the extent ... my memory is that generally the prediction models explain on average 20% of gene expression variation, so the true amount of variance explained could easily be five times higher than estimated). Furthermore, when an imputed gene expression is generated by a single-snp predictor model (or a model dominated by one snp), which again from memory is most gene expressions, how can you be confident the corresponding variance is that due to gene expression. To me, your analysis seems very similar to performing a two-GRM analysis, where one is constructed from all SNPs, and the other from just eQTLs. Even if eQTLs were no more important than average SNPs, we would still expect the eQTL grm to explain a sizable share of phenotypic variation (because it contains a sizable share of SNPs). Thus it seems sensible to perform an additional analysis, where you make these two GRMs (one from all snps, the other from SNPs used to predict any gene expression) ... if gene expressions do contribute large phenotypic variation, then we would expect the variance explained by the eqtl GRM to be a lot lower than the explained by your imputed gene expression GRM.

2 - The second application partitions variance explained into regulatory, DNAse1 and other SNPs, a more general version of Gusev et al 2015 (they include three GRMs, not two, and add in covariances). However later studies have found the analysis of Gusev et al tended to overestimate variance explained by functional categories (most notably, Gusev estimated DNAse1 explained 80% of SNP heritability, but more recent papers have estimated they explain closer to 30%). I can envisage using Core GREML could reduce the inflation, but I am not sure to what extent.

Despite these criticisms of the application, I still feel the method has value (hopefully someone else will find better applications). Further, I consider the prediction analysis (in particular, how it is used to support the results of application 1) very good.

Minor comments

3 - "Using extensive simulations, we show that CORE GREML outperforms the conventional GREML, providing unbiased estimates of variance and covariance components." In my view, this is an overstatement (I do not see how a simulation study can show a method [always] provides unbiased estimates)

4 - I cant find run times. I guess CORE REML takes days to analyze UKBB, but that it is no slower than standard GREML (at least once you have more than one GRM).

5 - L88 "from theUK biobank87data that are available to us (reference numb er 14575), including standing height," I think no need to say "including" (as you list all ten traits)

6 - L119 "yielded significant results 118for 19replicates out of 500,". Please state significance threshold used (I guess five percent)

Figure 2 - "Larger", not "large". Also, the final sentence is surely false? "The p-value is for the t-test statistic (df=8) under the null hypothesis that the slope of the regression line is zero". I suggest you replace zero with something like P<1e-16.

Signed Doug Speed


Reviewer #2 (Remarks to the Author):

Classic LMMs assume independence between random effects. Here, the authors proposed a new method, namely CORE GREML, that also considers the covariance between variance components. The authors performed simulations and showed that without considering the covariance term, the estimates might be biased. The authors performed genomic partitioning analyses using the UK biobank data and found significant correlations between the phenotypic effects of the regulatory regions and DHS for height and sitting height. The authors also performed genome-transcriptome partitioning using the imputed transcriptome data. They found a significant proportion of heritability contributed by transcriptome. They also found a significant correlation between the genome component and the transcriptome component for several UK biobank traits

I agree with the authors that it is important to consider the covariance terms. And the proposed CORE GREML method seems to be a natural extension of the original GREML method to this specific case. Also, the simulations and real-data analyses are comprehensive. So overall, I think the paper is well-motivated, well written, and technical sound. Please see my detailed comments below.

Major:

- Line 116-123: What is the significance threshold in the null simulation? I assume it is 0.05, but I could not find such information in the paper.

- Line 227: The authors mentioned that they dropped the non-significant covariance terms and refitted the model. Is that necessary? What will happen if they do not drop the non-significant component?

- For GREML-based method, a more popular way to address the LD- and MAF-dependent architecture seems to be stratifying the SNPs into different LD and MAF bins ( e.g., Yang et al. Nature Genetics, 2015), instead of having explicit assumptions on the genetic architecture. Have the authors considered that?

- For the users' benefit, it would be helpful to show the scalability of the method. I imagine it would be slower than the classical GREML, but I am not sure how much slower it is. For example, how long does it take to run on the full data with 91k samples?

- Line 434: In Eq (2), $var(y) = E[(g + e)^2]$, which is not equal to $(\sqrt{A} g + e)^2$ as written in the second term of Eq (2). The third term of Eq (2) makes sense though.

- Line 443: Similarly, In Eq (3), $var(y) = E[(g + e)^2]$ which is not equal to $(\sqrt{A} g + e)^2$ as written in the second term of Eq (3). Also like the last commnet, the third term of Eq (3) makes sense though.


Minor:

- Figure 2 caption: I don't understand the first sentence: " Imputed transcriptome and covariance between phenotypic effects of the genome and of the imputed transcriptome improve phenotypic prediction accuracy." Also, a typo "...Panel b. the large the..." --> "...Panel b. the larger the...".

**Author rebuttal, first version:**

Reviewers' comments:

**Reviewer #1 (Remarks to the Author):**

Lee et al propose Core GREML, which generalizes GREML by including covariance terms to allow for correlations between random effects. I see importance in the proposed method, especially (as the authors suggest) for multiomic analyses – ie, deciding the relative contributions of different classes of genetic variation. For example, we know that SNPs explain substantial variance of phenotypes, and that gene expressions also explain substantial. However, because snps and gene expressions are correlated, it is hard to know to what extent snps explain variance because they influence gene expression, or to what extent gene expressions appear to explain variance because they correlate with SNPs.

Major comments

The method seems accurate (mathematically, adding covariances is close to trivial, so existing solvers can be used to estimate variance components). However, I consider the two applications quite poor.

1 - The first (as described above) seeks to estimate the relative contributions of SNPs and gene expressions to phenotypes. A major shortcoming of this analysis is that, because (actual) gene expression data are not available for UKBB, the authors instead use imputed gene expression. These are obtained by first constructing simple (generally 1, 2 or 3 parameter) SNP-prediction models for gene expressions from a reference transcription database. The predicted gene expression tend to explain a relatively small fraction of gene expression variation and thus the final estimate of variance explained by gene expression will likely dramatically underestimate the true contribution of gene expression (the authors acknowledge under-estimation, but I think do not appreciate the extent ... my memory is that generally the prediction models explain on average 20% of gene expression variation, so the true amount of variance explained could easily be five times higher than estimated).

Response (R): We agree with the reviewer on that the phenotypic variance explained by the transcriptome would be underestimated in our study, given that the transcriptome is imputed, which we have acknowledged in the manuscript. However, we would like to point out that our intention of using the imputed transcriptome is not to accurately estimate variance explained by the transcriptome; but as a proxy of the transcriptome, i.e. 13.7% of gene expression variation on average (Gamazon et al, 2015, *Nat. Genet.* 47), to demonstrate the use of CORE GREML for genome-transcriptome partitioning analyses (discussed in L378-385). Raw gene expression data are likely to become abundant in the near future when one can obtain reliable estimates of phenotypic variance explained by the full gene expression variation using CORE GREML.

Furthermore, when an imputed gene expression is generated by a single-snp predictor model (or a model dominated by one snp), which again from memory is most gene expressions, how can you be confident the corresponding variance is that due to gene expression. To me, your analysis seems very similar to performing a two-GRM analysis, where one is constructed from all SNPs, and the other from just eQTLs. Even if eQTLs were no more important than average SNPs, we would still expect the eQTL grm to explain a sizable share of phenotypic variation (because it contains a sizable share of SNPs). Thus it seems sensible to perform an additional analysis, where you make these two GRMs (one from all snps, the other from SNPs used to predict any gene expression) ... if gene expressions do contribute large phenotypic variation, then we would expect the variance explained by the eqtl GRM to be a lot lower than the explained by your imputed gene expression GRM.

R: We thank the reviewer for the comment. We would like to clarify that the expression imputation for a given gene is based on multiple SNPs (see Gamazon et al, 2015, *Nat. Genet.* 47 for the standard transcriptome imputation protocol using PrediXcan), not a single SNP; and for all genes across 43 tissues, the imputation involves a total of 1,316,391 SNPs, i.e., the whole genome. We understand that a key concern raised by the reviewer is that the effects of the imputed transcriptome on phenotypes of a given trait might not be orthogonal to the effects of the SNPs that were used to impute the transcriptome. We performed two additional analyses to address this concern.

First, according to the reviewer’s comment, we constructed a GRM with the 1,316,391 SNPs that were used for the transcriptome imputation and denoted their effects as g_1_ in a model that expresses the phenotypes of a trait, y, as y = g_1_ + t + ε, where t is the effects of the imputed transcriptome and ε is residuals. If the imputed transcriptome has any effects on phenotypes that are orthogonal to g_1_ alone, this model would have a better fit than a reduced model with g_1_ only, i.e., y = g_1_ + ε. Based on the likelihood ratio test with one degree of freedom, y = g_1_ + t + ε had a better fit than y = g_1_ + ε for all traits except years of education and fluid intelligence (see Supplementary Table 6 below), indicating that t is orthogonal to g_1_ for most traits.

Second, we compared two models, y = g_0_ + g_1_ + ε and y = g_0_ + t + ε, where g_0_ denotes the phenotypic effects of 1,131,002 SNPs in our original genome-transcriptome partitioning analyses. It is noted that the set of SNPs for estimating g_0_ were ones that remained after applying our quality control criteria (L571-587), whereas the 1,316,391 SNPs for the transcriptome imputation (hence for estimating g_1_) were based on the transcriptome imputation protocol (L622-629; Gamazon et al., 2015, *Nat. Genet.* 47). The two sets of SNPs overlap by 716,636. If the two-GRM model (y = g_0_ + g_1_ + ε) is no different from the genome-imputed transcriptome model (y = g_0_ + t + ε), their likelihoods would be very similar. Contrary to this, we found that y = g_0_ + t + ε has a much better fit than y = g_0_ + g_1_ + ε for all traits except heel bone mineral density, years of education and fluid intelligence (see Supplementary Table 6 below), confirming that g_1_ and t are distinct for most traits. In addition, the results also indicate that y = g_0_ + t + ε is closer to the true underlying model than y = g_0_ + g_1_ + ε for most traits.

We have now included these additional analyses in the manuscript (see L205-229 in main text; supplementary materials).

Supplementary Table 6. The effects of the imputed transcriptome are orthogonal to the effects of SNPs used for the transcriptome imputation.

Note. **y** = phenotypes; **t** = the random effects of the imputed transcriptome; **g_0_** = the random effects of 1,074,458 SNPs for genome-transcriptome partitioning analyses presented throughout the main text; **g_1_** = the random effects of 1,316,391 SNPs that were used for the transcriptome imputation; ε = residuals. Log-likelihood_x_ = log-likelihood of model x, where x = a, b, c, or d; Δ log-likelihood_x-y_ = log-likelihood of model x – log-likelihood of model y, where x = a or b and y = c or d.

2 - The second application partitions variance explained into regulatory, DNAse1 and other SNPs, a more general version of Gusev et al 2015 (they include three GRMs, not two, and add in covariances). However later studies have found the analysis of Gusev et al tended to overestimate variance explained by functional categories (most notably, Gusev estimated DNAse1 explained 80% of SNP heritability, but more recent papers have estimated they explain closer to 30%). I can envisage using Core GREML could reduce the inflation, but I am not sure to what extent.

Despite these criticisms of the application, I still feel the method has value (hopefully someone else will find better applications). Further, I consider the prediction analysis (in particular, how it is used to support the results of application 1) very good.

R: Thanks for the comment. We agree that the genome-partitioning approach by Gusev et al is not perfect. The bias of variance estimates in this approach could be due to: 1) misspecification of annotation boundaries (e.g. causal variants not in a category but close to the boundary); 2) wrong model assumption about the underlying genetic architecture (i.e. heritability model); and 3) neglecting the covariance between random effects of annotation categories.

For 1), we would like to point out that the Gusev approach (top of Figure 2 in Gusev et al.) produced unbiased estimates of the percentage of SNP heritability explained by a given annotation category. It is the GWAS p-value enrichment approach that can produce false-positives for other categories that are in linkage disequilibrium with the causal category, even when they were not enriched (for which Finucane et al. (2015) has suggested a baseline model to remedy this problem). Since the term ‘enrichment’ can be confused with the p-value enrichment approach, we used the term ‘proportion of total genetic variance attributable to a given functional category’ instead. However, whether using partitioned SNP-heritability or the GWAS p-value enrichment approach (including a baseline model), estimates can be biased if the assumption on the underlying genetic architecture is wrong (i.e. #2).

For 2), we found that misspecification of the underlying genetic architecture in the estimation model biased CORE GREML estimation of variance components in general, but not the covariance term as shown in Supplementary Figure 4. Nonetheless, under any given genetic architecture, misspecification can be feasibly diagnosed by comparing the likelihood of estimation models that assume a wide range of possible genetic architectures. As shown in Supplementary Figure 5, differences in the likelihood of estimation models are highly indicative of deviations from the true underlying genetic architecture. In addition, given the results comparing GCTA model and LDAK model (Supplementary Tables 3 and 4), it is unlikely that the estimates are substantially inflated due to misspecification of heritability model, noting that estimates from two distinct heritability model are not far apart. Finally, the estimated proportion of DHS SNP-heritability over the total SNP-heritability is 0.4 on average, ranging from 0.19 to 0.69 (Supplementary Figure 9), which seems more reasonable than that of Gusev’s study (80% of the total SNP heritability).

For 3), this is the reason we propose CORE GREML. We showed that modelling covariance between the random effects of two categories may be necessary (e.g. Figure 6) to avoid biased estimates of variance components, when the correlation between random effects is not negligible.

Minor comments

3 - "Using extensive simulations, we show that CORE GREML outperforms the conventional GREML, providing unbiased estimates of variance and covariance components." In my view, this is an overstatement (I do not see how a simulation study can show a method [always] provides unbiased estimates).

R: We reworded our statement such that it is more specific about the type of bias that CORE GREML can avoid. “Using extensive simulations, we show that CORE GREML outperforms the conventional GREML, providing variance and covariance estimates free from bias due to correlated random effects” (see L6-8).

4 - I can’t find run times. I guess CORE REML takes days to analyze UKBB, but that it is no slower than standard GREML (at least once you have more than one GRM).

R: The computational requirements for fitting a model with two random effects using CORE GREML are outlined in Supplementary Table 8 below. Since CORE GREML uses the direct AI algorithm (Lee & Van Der Werf, 2006, *Genet Sel. Evol.* 38; Yang, Lee, Goddard, & Visscher, 2011, *Am. J. Hum. Genet.* 88), the computational efficiency is robust to increasing number of random effects including their covariance terms in the model. This information has now been included in the manuscript (see L555-558).

Supplementary Table 8. Computational resource requirements of CORE GREML for fitting a model with two random effects.

Note: All analyse were performed using mtg2.14, with each running on a single central processing unit at a speed between 1200 and 3200 MHz. Task duration is partitioned into time taken for reading in kernel matrices and computing time. RAM = random access memory. When using multiple CPUs with parallel computing, the computing time is expected to reduce in a curvilinear fashion.

5 - L88 "from the UK biobank data that are available to us (reference number 14575), including standing height," I think no need to say "including" (as you list all ten traits).

R: The sentence has been rephrased. See L88-91.

6 - L119 "yielded significant results 118 for 19 replicates out of 500,". Please state significance threshold used (I guess five percent)

R: The alpha level or the significance threshold used is 0.05. This piece of information has now been included in the revised manuscript (L117).

Figure 2 - "Larger", not "large". Also, the final sentence is surely false? "The p-value is for the t-test statistic (df=8) under the null hypothesis that the slope of the regression line is zero". I suggest you replace zero with something like P<1e-16.

R: The typo has been corrected accordingly. We have now revised the sentence regarding the p-value to avoid confusion. “A t-test with eight degrees of freedom was used to determine if the slope of the regression line differs from zero (see p-value on the left corner)” (P26 Figure 2 legend).

**Reviewer #2 (Remarks to the Author):**

Classic LMMs assume independence between random effects. Here, the authors proposed a new method, namely CORE GREML, that also considers the covariance between variance components. The authors performed simulations and showed that without considering the covariance term, the estimates might be biased. The authors performed genomic partitioning analyses using the UK biobank data and found significant correlations between the phenotypic effects of the regulatory regions and DHS for height and sitting height. The authors also performed genome-transcriptome partitioning using the imputed transcriptome data. They found a significant proportion of heritability contributed by transcriptome. They also found a significant correlation between the genome component and the transcriptome component for several UK biobank traits

I agree with the authors that it is important to consider the covariance terms. And the proposed CORE GREML method seems to be a natural extension of the original GREML method to this specific case. Also, the simulations and real-data analyses are comprehensive. So overall, I think the paper is well-motivated, well written, and technical sound. Please see my detailed comments below.

Major:

- Line 116-123: What is the significance threshold in the null simulation? I assume it is 0.05, but I could not find such information in the paper.

R: We thank the reviewer for this comment. The significance threshold is 0.05. We have now included this information in the manuscript (L117).

- Line 227: The authors mentioned that they dropped the non-significant covariance terms and refitted the model. Is that necessary? What will happen if they do not drop the non-significant component?

R: While it is common practice, reducing a model by removing non-significant parameters is not entirely necessary and has no consequences on parameter estimates. As shown in the table below, there are minimal differences in parameter estimates between the full and reduced models for height and sitting height. However, as expected, the standard errors of some parameter estimates are notably smaller for the reduced model than for the full model.

Parameter estimates by estimation model for height and sitting height.

- For GREML-based method, a more popular way to address the LD- and MAF-dependent architecture seems to be stratifying the SNPs into different LD and MAF bins ( e.g., Yang et al. Nature Genetics, 2015), instead of having explicit assumptions on the genetic architecture. Have the authors considered that?

R: We would like to thank the reviewer for this comment. We considered the possibility of partitioning genome-wide SNPs into subsets by LD structure and MAF bin and specifying a separate random-effect term for each subset in our model. However, this would inevitably result in many random-effect terms in the model, such that fitting CORE GREML would eventually become infeasible. We have mentioned this as a limitation in the manuscript (L418-425). Nonetheless, a misspecification of LD- and MAF-dependent architecture can be feasibly diagnosed by comparing the likelihood of estimation models that assume a wide range of possible architectures. As shown in Supplementary Figure 5, differences in the likelihood of estimation models are highly indicative of deviations from the true underlying genetic architecture.

- For the users' benefit, it would be helpful to show the scalability of the method. I imagine it would be slower than the classical GREML, but I am not sure how much slower it is. For example, how long does it take to run on the full data with 91k samples?

R: The computational requirements for fitting a model with two random effects using CORE GREML are outlined in Supplementary Table 8 below. It is noted that CORE GREML uses the direct AI algorithm (Lee & Van Der Werf, 2006, *Genet Sel. Evol.* 38; Yang, Lee, Goddard, & Visscher, 2011, *Am. J. Hum. Genet.* 88), the computational efficiency of which is robust to increasing number of the random effects including their covariance in the model. This information has now been included in the manuscript (see L555-558).

Supplementary Table 8. Computational resource requirements of CORE GREML for fitting a model with two random effects.

Note: All analyse were performed using mtg2.14, with each running on a single central processing unit (CPU) at a speed between 1200 and 3200 MHz. Task duration is partitioned into time taken for reading in kernel matrices and computing time. RAM = random access memory. When using multiple CPUs with parallel computing, the computing time is expected to reduce in a curvilinear fashion.

- Line 434: In Eq (2), $var(y) = E[(g + e)^2]$, which is not equal to $(\sqrt{A} g + e)^2$ as written in the second term of Eq (2). The third term of Eq (2) makes sense though.

R. We have removed the 2^nd^ term in Eq(2) and revised the text accordingly (P15)

- Line 443: Similarly, In Eq (3), $var(y) = E[(g + e)^2]$ which is not equal to $(\sqrt{A} g + e)^2$ as written in the second term of Eq (3). Also like the last comment, the third term of Eq (3) makes sense though.

R. We have removed the 2^nd^ term in Eq(3) and revised the text accordingly (P15).

Minor:

- Figure 2 caption: I don't understand the first sentence: " Imputed transcriptome and covariance between phenotypic effects of the genome and of the imputed transcriptome improve phenotypic prediction accuracy." Also, a typo "...Panel b. the large the..." --> "...Panel b. the larger the...".

R: We have revised the Figure 2 title and corrected the typo in the legend. “Improved phenotypic prediction accuracy when accounting for the effects of the imputed transcriptome (a) and the covariance between the effects of the genome and the imputed transcriptome (b)” (see P26 Figure 2 legend).

**Reviewer comments, second version:**

Reviewer #1 (Remarks to the Author):

1a - I think a bit hopeful to think gene expression data for datasets containing thousands of unrelated individuals will be available any time soon. But as I said previously, the method is more important than the applications.

1b - Thank you for the extra analysis. You did what I asked, but when I suggested, I had not realised the differences in SNP-sets, which mean that this extra analysis only offers partial reassurance. It seems now that your analyses generally had about 1.3M snps after QC, but to predict the transcriptome, you used a different set of 1.3M ... those reported by Gamazon ... of which about half are common to the two steps. It is concerning that you therefore used about 600k snps to predict gene expressions, even though you considered these too poor quality to include in your main analysis. Further, this makes the analyses less elegant. I thought you were comparing the basic grm (which is constructed from about 1.3M snp) to basic grm plus transcriptome grm (constructed from about 40k new predictors, each a linear combination of the 1.3M snps). Instead you are comparing basic grm, to basic grm plus a transcriptiome grm (constructed from about 40k new predictors, each a linear combination of about 700k new snps plus 600 other snps). So I thought you were showing that model fit is improved by using each of the 1.3M snps plus combinations of these snps. But instead, it is unclear how much the improvement is due to adding linear combinations or the 1.3M snps, or because you are introducing new information fro 600k snps.

So to try and restate. You coami that your extra analyse shows t is orthogonal to g1 for most traits (well really, has a significant orthogonal proportion). But this could be because the transcription is constructed from a substantial number of extra snps (of possibly dodgy quality). You could improve by either increasing your starting set of snps from 1.3M to 2M (to include all those of gamazon) or reducing gammazon snps to those in your 1.3M

2 - Thanks for the thorough explanation

Thanks also for dealing with the minor comments

Doug Speed

Ps, the shortness of this review partly reflects it is the fourth time I have given comments on this paper! I believe even Hercules had his limits ...


Reviewer #2 (Remarks to the Author):

I would like to thank the authors for their efforts. The revision has successfully addressed all my concerns, and I do not have additional comments.

Martin Jinye Zhang

**Author rebuttal, second version:**

Reviewer #1 (Remarks to the Author):

1a - I think a bit hopeful to think gene expression data for datasets containing thousands of unrelated individuals will be available any time soon. But as I said previously, the method is more important than the applications.

Response: We thank the reviewer for acknowledging again the importance of CORE GREML method.

1b - Thank you for the extra analysis. You did what I asked, but when I suggested, I had not realised the differences in SNP-sets, which mean that this extra analysis only offers partial reassurance. It seems now that your analyses generally had about 1.3M snps after QC, but to predict the transcriptome, you used a different set of 1.3M ... those reported by Gamazon ... of which about half are common to the two steps. It is concerning that you therefore used about 600k snps to predict gene expressions, even though you considered these too poor quality to include in your main analysis. Further, this makes the analyses less elegant. I thought you were comparing the basic grm (which is constructed from about 1.3M snp) to basic grm plus transcriptome grm (constructed from about 40k new predictors, each a linear combination of the 1.3M snps). Instead you are comparing basic grm, to basic grm plus a transcriptome grm (constructed from about 40k new predictors, each a linear combination
of about 700k new snps plus 600 other snps). So I thought you were showing that model fit is improved by using each of the 1.3M snps plus combinations of these snps. But instead, it is unclear how much the improvement is due to adding linear combinations or the 1.3M snps, or because you are introducing new information from 600k snps.

So to try and restate. You claim that your extra analysis shows t is orthogonal to g1 for most traits (well really, has a significant orthogonal proportion). But this could be because the transcription is constructed from a substantial number of extra snps (of possibly dodgy quality). You could improve by either increasing your starting set of snps from 1.3M to 2M (to include all those of gamazon) or reducing gamazon snps to those in your 1.3M

R: We thank the reviewer for the thorough explanation of his concerns, and agree that the comparison should be made between the basic grm (which is constructed from about 1.3M SNPs) and basic grm plus transcriptome grm (constructed from about 40k new predictors, each a linear combination of the same set of 1.3M SNPs). After thorough checks of the Supplementary Table 6, we found that the suggested model comparison has already been conducted (**y** = **g_1_** + **t** + **ε** VS. **y** = **g_1_** + **ε** where **g_1_** and **t** both were based on the same set of 1.3M SNPs). We realised that that we have not explained the detail of two models presented in Supplementary Table 6 clearly enough, such that the reviewer has been confused about the sets of SNPs used for the two models. We would like to clarify the detail below (and also in the legend of Supplementary Table 6; highlighted in red):

Model #1 (shown as (a) in Supplementary Table 6): **y** = **g_1_** + **t** + **ε,** where **g_1_** is based on 1.3M SNPs, and **t** is based on gene expressions that are imputed using the same set of 1.3 M SNPs for **g_1_**. Please note that the imputation strictly follows the well-established imputation protocol (Gamazon et al, 2015, *Nat. Genet.* 47).

Model #2 (shown as (d) in Supplementary Table 6): **y**= **g_0_** + **t** + **ε,** where **g_0_** is based on 1.1M SNPs, and **t** is based on gene expressions that are imputed using 1.3M SNPs (as in model #1), which have 700K SNPs in common with the 1.1M SNPs for **g_0_**.

We agree with the reviewer that 1) comparing **y** = **g_0_** + **t** + **ε** with **y** = **g_0_** + **ε** does only offer partial reassurance**,** since **g_0_** is based on 1.1M SNPs and **t** is based on a linear combination of 700k SNPs in common with **g_0_** plus 600K extra SNPs; and 2) to show that SNP effects and transcriptomic effects are orthogonal, GRM and transcriptome imputation should be based on the same set of SNPs. Hence, we fitted model #1, as requested by the reviewer, and showed that this model had a better fit than **y** = **g_1_** + **ε** for 8 out of the 10 traits considered (shown in Supplementary Table 6 as (a) vs. (b) for our primary analysis).

As a secondary analysis, we also compared models **y** = **g_0_**+ **g_1_**+ **ε** (c in Supplementary Table 6) and **y** = **g_0_**+ **t** + **ε** (d). We showed that the model-fit of (d) was much better than (c) for many traits, confirming again that **g_1_** and **t** are orthogonal. Once again, we would like to emphasize that **g_1_** and **t** were based on the same set of SNPs (1.3M). We also carefully checked other possible model comparisons, including **y** = **g_0_**+ **g_1_**+ **ε** (c) VS. **y** = **g_1_**+ **t** + **ε** (a) and **y** = **g_1_**+ **g_1_**+ **ε** VS. **y** = **g_1_**+ **t** + **ε**. However, the former (i.e., **g_0_** VS. **t**) is not based on the same set of SNPs, and the latter includes an invalid model.

Supplementary Table 6. The effects of the imputed transcriptome on phenotypes are orthogonal to the effects of SNPs used for the transcriptome imputation.

Notes. 1. **y** = phenotypes; **t** = random effects of the imputed transcriptome; **g_0_** = random effects of 1,074,458 SNPs for genome-transcriptome partitioning analyses presented throughout the main text; **g_1_** = random effects of 1,316,391 SNPs used for the transcriptome imputation; **ε** = residuals. Log-likelihood_x_ = log-likelihood of model x, where x = a, b, c, or d; Δ log-likelihood_x-y_ = log-likelihood of model x – log-likelihood of model y, where x = a or b and y = c or d. 2. It is important to distinguish the SNP sets involved for different variance components of the models. The kernel matrix for **t** is based on gene expressions imputed using 1,316,391 SNPs, which is the same set of SNPs used to construct the kernel matrix for **g_1_**. This set of SNPs only has ~ 600K in common with the 1,074,458 SNPs used to construct the kernel matrix for **g_0_**. In our primary analysis (model a VS. model b), we show that model a has a better fit than model b for eight traits, even though **t** and **g_1_** are based on the same set of SNPs. Hence, **t** is orthogonal to **g_1_** for most traits. In our secondary analysis (model c VS. model d), we show that model d has a better fit than model c for seven traits—once again—despite the fact that **t** and **g_1_** are based on the same set of SNPs. This confirms that **t** and **g_1_** are distinct for most traits

The reviewer also suggested that “(we) could improve by either increasing (our) starting set of snps from 1.3M to 2M (to include all those of Gamazon)…”. We would like to clarify that the 2M SNPs in Gamzaon is the starting set for training their transcriptome imputation model. The GTEx v7 model (2018-01-08 release; <http://predictdb.org/>) provided effect estimates for 1.3M SNPs only, which are subsequently used for the transcriptome imputation in our study.

In addition, the reviewer also expressed the concern that “ …(we) used about 600k SNPs to predict gene expressions, even though you considered these too poor quality to include in (our) main analysis (i.e., for basic GRM)”. We would like to point out the followings:

1) These 600K SNPs (together with other 700K SNPs for the transcriptome imputation) have all passed our quality control, such that they all have an INFO score > 0.6, a minor allele frequency > 0.01, a p-value for the Hardy-Weinberg test > 0.0001, and missingness < 0.05 (L625-626).
2) The reason that these 600K SNPs are used for the transcriptome imputation is that we strictly followed the well-established transcriptome imputation protocol (Gamazon et al, 2015, *Nat. Genet.* 47; > 600 citations since 2015), which used HapMap Phase II (instead of Phase III) SNPs. Excluding these 600K SNPs may substantially compromise the accuracy of transcriptome imputation.

Finally, to rule out the possibility that SNP selection (i.e., 1.3M vs. 1.1M SNPs) affects CORE GREML estimation, we have newly performed a sensitivity analysis, in which HapMap phase II 1.3M SNPs were used for both GRM and transcriptome imputation when estimating genome-transcriptome correlation with CORE GREML. Supplementary Table 7 below displays estimates of the covariance term by SNP set. The correlation between the two sets of covariance estimates is 0.92 and the correlation between the two sets of -log10(p-value) is 0.97. In absolute terms, though, both covariance estimates and the significance level reduced when using HapMap phase II SNPs than when using HapMap phase III SNPs. Nonetheless, significant genome-transcriptome covariances for standing height, sitting height, and diastolic blood pressure remain. Therefore, regardless of which SNP set—HapMap phase II or HapMap phase III—is used, our main conclusion about the genome-transcriptome covariance still holds. We have now included this information in the main text (L269-275) and supplementary materials (Supplementary Table 7).

Supplementary Table 7. Genome-Transcriptome covariance estimates based on HapMap phase II and HapMap phase III SNPs.

Note: p values are based on likelihood ratio tests with one degree of freedom that compare GREML with CORE GREML to detect the genome-transcriptome covariance term, i.e., $\sigma_{g0 t}$ or $\sigma_{g1 t}$. The kernel matrix for **g_1_** is based on 1,316,391 HapMap phase II SNPs, and the kernel matrix for **g_0_** is based on 1,074,458 HapMap phase III SNPs, which has ~600K SNPs in common with the HapMap phase II SNPs for **g_1_**. The kernel matrix for **t** is based on gene expressions imputed using the same set of 1,316,391 HapMap phase II SNPs as for **g_1_**.

2 - Thanks for the thorough explanation

R: We are pleased by this acknowledgement.

Thanks also for dealing with the minor comments

R: Thanks for the comments that help us improve the manuscript.

Reviewer #2 (Remarks to the Author):

I would like to thank the authors for their efforts. The revision has successfully addressed all my concerns, and I do not have additional comments.

R: We appreciate the reviewer for the helpful and comprehensive comments.

**Reviewer comments, third version:**

**Author rebuttal, third version:**
